# Network models for bridging denoising and identifying spatial domains of spatially resolved transcriptomics

**DOI:** 10.1371/journal.pcbi.1013867

**Published:** 2026-01-13

**Authors:** Haiyue Wang, Wensheng Zhang, Zaiyi Liu, Xiaoke Ma

**Affiliations:** 1 School of Physics and Electronics, Shandong Key Laboratory of Medical Physics and Image Processing, Shandong Normal University, Jinan, China; 2 School of Computer Science and Technology, Xidian University, Xi’an, Shaanxi, China; 3 School of Computer Science and Cyber Engineering, Guangzhou University, Guangzhou, China; 4 Department of Radiology, Guangdong Provincial People’s Hospital (Guangdong Academy of Medical Sciences), Southern Medical University, Guangzhou, China; 5 Guangdong Provincial Key Laboratory of Artificial Intelligence in Medical Image Analysis and Application, Guangzhou, China; 6 Key Laboratory of Smart Human-Computer Interaction and Wearable Technology of Shaanxi Province, Xidian University, Xi’an, China; Jilin University, CHINA

## Abstract

Spatially resolved transcriptomics (SRT) enables the simultaneous capture of gene expression profiles and spatial localization, providing valuable insights into tissue architecture. However, the preservation of spatial information requires additional experimental procedures, which often introduce substantial technical noise. Existing methods typically perform denoising and spatial domain identification in separate steps, leading to suboptimal performance and limiting their applicability. To address this limitation, we propose an integrative network model, stACN ( **s**patial **t**ranscriptomics **A**ttribute **C**ell **N**etwork), that jointly denoises gene expression data and identifies spatial domains in SRT. Specifically, stACN first learns clean dual cell networks using a graph noise model, and then derives compatible cell features through joint tensor decomposition of the denoised networks. Experimental results demonstrate that stACN effectively enhances data quality, as measured by clustering agreement with reference annotations (Adjusted Rand Index, ARI), and facilitates spatial domain analysis in SRT datasets.

## Author summary

Spatially resolved transcriptomics (SRT) simultaneously captures gene expression and spatial localization within intact tissues, providing a powerful tool for studying tissue organization and disease progression in fields such as developmental biology and oncology. However, the additional experimental procedures required to retain spatial context often introduce substantial technical noise, resulting in data that are typically sparse and noisy, thereby posing significant challenges for downstream analysis. To address these issues, we propose a network-based integrative model, stACN, for denoising and identifying spatial domains in SRT data by leveraging the topological structure of cell networks. Specifically, stACN constructs spatial and expression graphs through representation learning, denoises the data via graph-based modeling, performs joint feature learning through matrix decomposition, and identifies spatial domains by exploiting the structure of the cell affinity graph. Extensive experiments across diverse SRT platforms demonstrate that stACN effectively delineates spatial domains, identifies domain-specific gene markers, and generalizes well across datasets. These results highlight the potential of stACN as a robust framework for the integrated analysis and denoising of SRT data.

## Introduction

Biological tissues execute their functions by simultaneously utilizing the spatial and expression contexts of cell types, which are crucial for characterizing the microenvironment within tissues [1–3]. Spatially resolved transcriptomics (SRT) measures gene expression in tissues while preserving the spatial location of each sequencing unit (referred to as “spots” in barcode-based technologies and “cells” in imaging-based technologies). Typical SRT platforms include PIXEL-seq [4], Stereo-seq [5], Slide-seq [6,7], and 10 × Visium [8], providing unprecedented opportunities to reveal tissue structure and function in a spatial context.

Identifying spatial domains that are continuous regions with consistent expression profiles is a prominent task for analysis of SRT data [9,10], which corresponds to the typical clustering problem that divides cells (spots) into highly similar groups. However, it is highly non-trivial for spatial domain detection because it simultaneously balances spatial and expression information. Fortunately, great efforts are devoted to this issue because of its significance [11–17]. Based on the use of spatial and expression information, existing algorithms can be roughly divided into two categories. The first group relies solely on expression profiles, whereas the second group integrates both spatial and expression information to identify spatial domains. Specifically, the most intuitive strategy for non-spatial algorithms is to execute clustering on expression of cells with various methods, such as K-means [11], Louvain [12], and algorithms for single-cell RNA-seq (scRNA-seq) [13–15,17]. Although these algorithms are popular for their simplicity, they are inapplicable because of the undesirable performance of ignoring spatial information.

Luckily, many algorithms [18–29] are developed to address this limitation by integrating spatial location and expression of units for the identification of spatial domains because spatial information plays an indispensable role in domain delineation. The key difference among these algorithms lies in their strategies for integrating expression and spatial information. For example, BayesSpace [18] and Giotto [19] adopt Bayesian and hidden Markov random field (HMRF) models to encourage the nearest cells with similar expression profiles belonging to the same spatial domains, where spatial locations are used as a priori information, respectively. However, these approaches perform well only when the structure of spatial domains is distinct, which cannot usually be satisfied because the heterogeneity of tissues results in a complicated structure of spatial domains. To overcome this issue, stLearn [20] leverages the identification of spatial domains by integrating histological images, gene expression, and spatial coordinates of cells, where morphological features of images provide complementary information.

However, these algorithms fail to capture indirect relations among cells for feature learning. The network model provides an alternative, which significantly improves the quality of features with various strategies [30–32]. For example, STAGATE [21] first learns the cell similarity graph via integrating expression and spatial information, and obtains the low-dimensional latent embedding of the graph with a graph attention auto-encoder, which significantly improves the performance of spatial domain identification. MNMST [22] employs a multi-layer network to identify spatial domains by analyzing cell network topology, improving precision and interpretability. SpaGCN [23] employs a graph convolutional network to learn a deep representation of cells, whereas SEDR [24] utilizes a deep auto-encoder network for learning representations of cells by simultaneously embedding spatial information. DeepST [33] integrates expression, spatial location, and morphological information to construct a cell network by setting correlations of spatially adjacent spots as weights, and a graph neural network (GNN) is employed to obtain cell features for spatial domain identification. SOTIP [34] incorporates gene expression profiles, micro-environments, and interrelationships of cells as a unified graph, whereas CCST [35] adopts a graph convolutional network to learn complicated cell interactions across tissues. SpaceFlow [36], BANKSY [37], and GraphST [38] are devoted to learning discriminative features of cells with sophisticated tools, such as self-supervised contrastive learning. These algorithms prove that networks of cells provide complementary information for expression and spatial information, thereby enhancing the accuracy of algorithms for spatial domain identification.

However, current methods for spatial domain identification implicitly hypothesize that SRT data are clean because they directly perform feature learning and spatial domain identification with the raw data. In practice, noise exists in sequencing data (for example, drop-out events [39,40] bring in noise in single-cell RNA-sequencing (scRNA-seq) data), which significantly affects the performance of algorithms for downstream analysis. Great evidence proves that severe noise also exists in SRT data, largely arising from the technical limitations of sequencing each unit. Furthermore, the delicate procedures required to preserve spatial information during sequencing can introduce additional noise [18]. Thus, denoising SRT data is the first step for spatial domain identification since noise masks the structure of domains to a large extent. For instance, Sprod [41] integrates physical locations and pathological tissue organization to perform denoising, whereas MIST [42] accomplishes denoising for regions with multiple cell types with low-rank matrix completion. SpotGF [43] leverages optimal transport to denoise SRT data, filtering out widespread expression genes while preserving raw sequencing data. These algorithms significantly enhance the performance of analyzing SRT data, demonstrating that denoising dramatically empowers SRT technologies for biomedical discoveries.

But, current efforts for denoising of SRT are really limited because there are still many unsolved problems on the strategies, models, and conditions for denoising. On the strategy issue, existing studies typically address noise by either neglecting it or simply removing noise as a pre-processing procedure, i.e., denoising and feature learning are independent. It should be noted that this independent noise removal strategy struggles to balance denoising with downstream tasks, such as spatial domain identification, resulting in undesirable performance. On the model issue, available algorithms model the noise of SRT data by manipulating spatial and expression features of cells, failing to precisely characterize complicated noise because they ignore heterogeneity and the intrinsic relation of cells. On the condition issue, current algorithms denoise SRT data by integrating multi-modal data [41], assuming that noise can be modeled and removed using additional information from sample-matched transcriptional, morphological, and spatial data. However, such preconditions cannot always be satisfied in practice due to the complexity of data collection. Thus, there is a critical need for denoising of SRT data, which can overcome these problems from the strategy, model, and condition perspectives.

**Fig 1:**
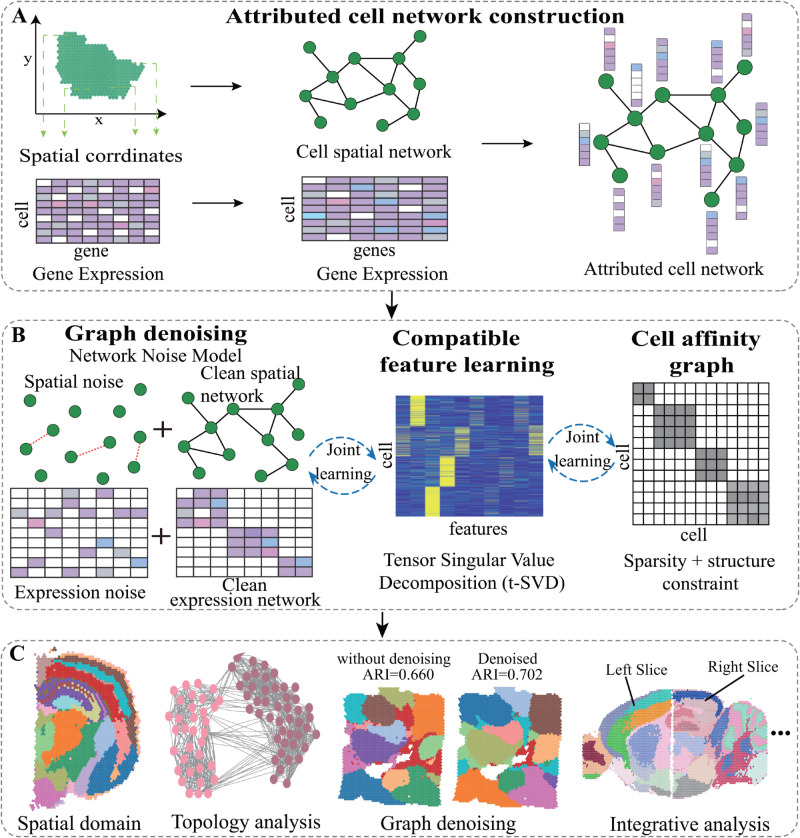
stACN is composed of four procedures, i.e., attributed cell network construction, graph denoising with network noise model, compatible feature learning, and downstream analysis. **(A)** The first procedure converts SRT data into an attributed cell network, graph denoising characterizes and removes noise of cells with network noise model. **(B)** The second procedure employs tensor singular value decomposition (t-SVD) by jointly factorizing the clean spatial and expression cell networks to extract compatible features of cells, which simultaneously preserves topological structure of spatial and expression graphs. **(C)** And, the downstream analysis of SRT data is performed by exploiting structure of cell affinity graph learned from compatible features of cells. Here, “cell” refers to a measurement unit in SRT data, i.e., either a single cell or a spot, depending on the dataset.

To address these problems, we bridge denoising and domain identification of SRT data (stACN) with an integrative framework by exploiting the topological structure of cell networks. Fig 1 illustrates the four procedures of stACN, including attributed cell network construction, graph denoising, compatible cell feature learning, as well as downstream analysis. On the strategy issue, stACN performs graph denoising of SRT data with joint learning, where graph denoising, feature learning, and spatial domain identification are incorporated into an overall framework. In this case, denoising is associated with the analysis of SRT data, rather than a pre-processing procedure, thereby improving the performance of denoising since downstream tasks guide denoising. On the model issue, stACN employs a graph denoising model to learn clean dual cell networks for SRT data, where the noise of cells is explicitly characterized and modeled. In this case, indirect topological structure relations of cell networks are fully exploited, which provides a better way to characterize complicated non-linear noise in SRT data. Moreover, graph denoising also facilitates the learning of compatible and interpretable features of cells by jointly factorizing the clean dual cell networks. On the condition issue, stACN performs graph denoising only by exploiting spatial and expression data without incorporating additional omic data, which can be directly applied to any SRT data. Extensive experiments on various data from different platforms and tissues demonstrate that stACN not only enhances the performance of analyzing SRT data but also serves as an effective tool for denoising SRT data.

## Results

### Overview of stACN

For the sake of convenience, we use cells to represent measurement units in spatial transcriptomics, such as spots in the 10× Visium platform, which can be interchanged freely.

stACN models and characterizes the structure of noisy SRT data by learning compatible cell features from clean cell networks with graph denoising. The design of stACN is shown in Fig 1, which is composed of four major procedures, i.e., attributed cell network construction, graph denoising with network noise model, compatible feature learning, and downstream analysis. On the construction of the attributed cell network construction issue (Fig 1 **A**), expression profiles of cells are normalized with library size (SCANPY [44]). And, we only keep these highly variable genes (3000 in total) that are expressed more than 10 spots with Seurat [45]. The expression data is augmented with BANKSY [37]. Then, an attributed cell network is constructed with edge weights as the spatial distance of cell pairs, and expression profiles as attributes of the vertices.

On the graph denoising issue, current algorithms remove noise in expression profiles of cells by leveraging spatial and pathological information in SRT data, which ignore relations among cells with the underlying hypothesis that cells are independent. Actually, this assumption deviates from expectation since cells within the same domains are highly related, thereby failing to model and remove complicated nonlinear noise. To address these issues, stACN performs graph denoising by jointly learning clean spatial and expression dual networks with a network noise model, which assumes that the observed networks are composed of the truth and noise components (Fig 1 **B**, Section of Methods). Specifically, stACN learns the cell network by manipulating the topological structure of the attributed network, and jointly removing noise on the edges of the cell spatial and expression networks with a low-rank constraint. Graph denoising of stACN results in three main advantages. First, it transforms expression and spatial information into two homogeneous networks, which significantly reduces the heterogeneity of SRT data. Second, it removes noise at the network level, rather than at the cell level, where the complicated nonlinear noise is effectively characterized and eliminated since indirect relations among cells are fully exploited. Third, stACN simultaneously performs denoising for the cell spatial and expression networks, which implicitly improves relations of spatial and expression networks, paving the way to learn cell features.

On the compatible feature learning issue, available methods either independently learn spatial and expression features of cells and concatenate them for downstream analysis or obtain discriminative features of cells using deep learning, such as a graph convolutional network (GCN), by fully exploiting the topological structure of cell networks. Even though these algorithms achieve excellent performance, they are criticized for failing to preserve the compatibility and interpretability of cell features. To address this issue, stACN learns cell features by integrating the clean cell spatial and expression networks via tensor decomposition, which projects cells into a unified tensor space (Fig 1 **B**). In detail, the clean cell dual networks are projected into a shared subspace, in which spatial and expression information are jointly coordinated, thereby enhancing compatibility of features. Furthermore, cell features are learned via t-SVD (tensor singular value decomposition), which improves the interpretability of features.

On the downstream analysis issue, stACN automatically learns an affinity graph of cells with the compatible cell features, which is accomplished by self-representation learning with sparse and structural constraints. In this case, biological structures, such as spatial domains, are well characterized by the topological structure of the cell affinity graph. The downstream analysis includes spatial domain detection, trajectory inference, and identification of spatially functional genes using data generated from various platforms and tissues (Fig 1 **C**). Moreover, stACN jointly learn all these procedures, including graph denoising, compatible feature learning, and affinity graph construction, which are formulated as a constrained optimization problem (Section Method), which dramatically facilitates users with limited knowledge in machine learning.

To the best of our knowledge, stACN is the first graph-based model for denoising SRT data, which jointly learns compatible and interpretable features of cells by integrating spatial and expression cell networks. It removes complicated noise at the network level, improving the reliability of cell networks. Furthermore, stACN is an end-to-end and efficient learning algorithm, which not only facilitates the analysis of SRT but also serves as the pre-processing step for denoising SRT data.

### stACN significantly improves compatibility of cell features

**Fig 2:**
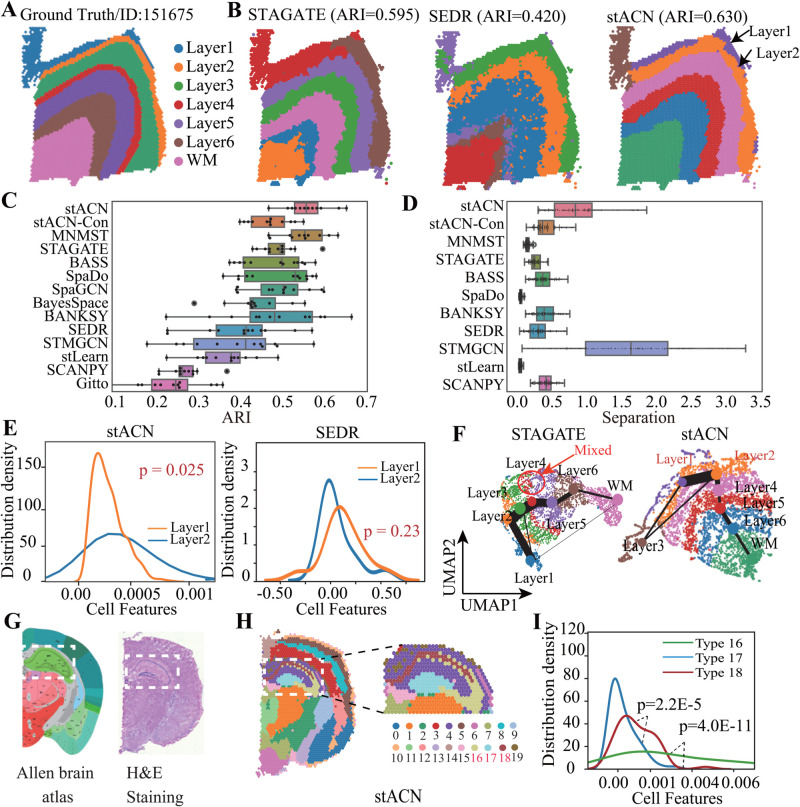
stACN precisely learns compatible features for spatial domains in brain tissues. **(A)** Annotation of layers for slice 151675 in DLPFC data, it is composed of six cortical layers from L1 to L6, as well as WM (white matter) [46]. **(B)** Visualization of spatial domains identified by various algorithms for slide 151675 of DLPFC dataset. **(C)** ARIs of various algorithms for identifying spatial domains in DLPFC dataset, where x-axis represents ARI (the center line, box limits and whiskers denote the median, upper and lower quartiles, and 1.5 × interquartile range, respectively). **(D)** Distributions of separation of spatial domains identified by various algorithms for all slice in DLPFC dataset, where separation is sum of distance among centers of domains. **(E)** Distribution density estimation between Layer1 and Layer2 with cell features learned by stACN (left) and SEDR (right), respectively, where the x-axis denotes cell features, and the Kolmogorov-Smirnov test is for significance. **(F)** UMAP visualizations and PAGA graphs of domains for slide 151675 obtained by STAGATE (left), and stACN embeddings (right), respectively. **(G)** Anatomical Allen Mouse Brain Atlas (left, https://atlas.brain-map.org/), H&E staining generated from raw data (right), and **(H)** spatial domains identified by stACN, where white dashed box denotes the cornu ammonis and dentate gyrus areas in the coronal portion. **(I)** Distribution density estimation among Type 16, 17 and 18 with cell features learned by stACN, and Kolmogorov-Smirnov test is for significance.

We first evaluate the performance of stACN in spatial domain identification and then assess the quality of cell features. The human dorsolateral prefrontal cortex (DLPFC) dataset (10 × Visium platform) [46] is selected as a benchmark, which consists of 12 slices, and each of them depicts the four or six layers of the human dorsolateral prefrontal cortex and white matter (WM) (Fig 2 **A**, Table A in S1 Text). Twelve state-of-the-art algorithms are deliberately chosen as baselines, including SCANPY [44], STAGATE [21], stLearn [20], BayesSpace [18], SpaGCN [23], SEDR [24], Giotto [19], BASS [47], SpaDo [48], MNMST [22], STMGCN [49], and BANKSY [37], to validate performance of stACN. SCANPY is a traditional method for spatial domain identification by ignoring spatial information. SEDR and stLearn are feature-based algorithms for spatial domains, where stACN also aims to learn compatible features of cells. STAGATE, SpaGCN, MNMST, and STMGCN are selected since they are typical network-based approaches, where stACN also adopts a network model. Other baselines are selected because of their excellent performance in analyzing spatial transcriptomics data.

Since spatial domains in the DLPFC are known, the performance of algorithms is quantified using the adjusted Rand index (ARI) and normalized mutual information (NMI; Methods section). Parameter selection indicates that stACN is robust (Section D and Fig A in S1 Text). The performance of various algorithms for slice 151675 in the DLPFC dataset is visualized in Fig 2 **B** (Fig B–Fig D in S1 Text), where stACN outperforms the baselines. Specifically, ARI of stACN is 0.630, compared with 0.595 and 0.420 for STAGATE and SEDR, respectively. STAGATE is inferior to stACN because it treats spatial information merely as complementary to expression profiles, failing to balance spatial and expression information effectively. Two factors explain why stACN outperforms the baselines. First, stACN jointly performs graph denoising for cell spatial and expression networks, thereby improving the quality of clean cell networks. Second, stACN learns compatible features of cells, providing a better way to characterize the structure of spatial domains. To further assess the spatial coherence of the learned embeddings, we compute Moran’s I, a global measure of spatial autocorrelation. Higher Moran’s I values indicate stronger spatial autocorrelation, meaning that neighboring cells tend to have similar embeddings. stACN achieves a Moran’s I of 0.668, compared with 0.782 and 0.321 for STAGATE and SEDR, respectively. These results demonstrate that stACN effectively balances spatial smoothness and expression variability, achieving strong spatial coherence and accurately characterizing spatial domains.

To check whether stACN is sensitive to slice 151675, we apply these algorithms to all 12 slices of the DLPFC dataset, and distributions of ARIs of various algorithms are described in Fig 2 **C** (Fig B–Fig D in S1 Text). It demonstrates that spatial domains identified by stACN are more consistent with manual annotation of DLPFC and the definition of cortical stratification of brain tissues than baselines. Specifically, ARI of stACN is 0.559 ± 0.049 (mean ± standard deviation), whereas it is 0.495 ± 0.042 (STAGATE), 0.494 ± 0.069 (SpaGCN), 0.435 ± 0.104 (BayesSpace), 0.415 ± 0.099 (SEDR), 0.250 ± 0.078 (Giotto), 0.380 ± 0.067 (stLearn), 0.271 ± 0.041 (SCANPY), 0.553 ± 0.073 (MNMST), 0.385 ± 0.111 (STMGCN), 0.489 ± 0.078 (SpaDo), 0.480 ± 0.120 (BANKSY), and 0.478 ± 0.070 (BASS), respectively. In addition to ARI, we evaluate clustering performance using NMI (Fig E in S1 Text). Consistent with ARI results, stACN achieves the highest NMI among all methods, further confirming the robustness of its spatial domain identification. These results show that spatial information is critical for spatial domains since SCANPY is inferior to others. stACN, MNMST, and STAGATE outperform other baselines, demonstrating that the network model is promising for spatial domain identification. Furthermore, the superiority of stACN and STAGATE also indicates that denoising is critical for spatial domain identification since SRT data suffer from severe noise, which is consistent with the assertion in Ref. [41]. Overall, network-based algorithms are superior to feature-based approaches for spatial domain identification, demonstrating that spatial domains can also be effectively characterized with the topological structure of cell networks. Then, we further investigate whether compatibility of cell features is also a critical factor for spatial domains by proposing a variant of stACN (called stACN-Con), which independently learns cell features for spatial and expression networks, respectively, and concatenates them to perform spatial domain identification. Interestingly, the performance of stACN-Con dramatically decreases (0.470±0.046), demonstrating that compatible features are promising for modeling spatial domains.

Next, we evaluate the spatial domains obtained by various algorithms using qualitative clustering indices, including separation and compactness. Separation is defined as the average distance among the centers of domains, while compactness is the average distance of each cell to the center of its domain. Fig 2 **D** shows the distribution of separation of spatial domains across all slices in the DLPFC dataset. Domains obtained by STMGCN are well separated (1.608 ± 0.791), followed by stACN (0.932 ± 0.483). The distribution of compactness further indicates that stACN achieves a favorable balance between separation and compactness (Fig A in S1 Text). Moreover, stACN-Con performs worse than stACN in both metrics (separation: 0.932 ± 0.483 vs. 0.431 ± 0.204; compactness: 0.206 ± 0.101 vs. 0.274 ± 0.138). Notably, the separation and compactness obtained by MNMST are small because it learns features of spots in a compact feature space.

Interestingly, stACN successfully delineates the Layer1 and Layer2 cortical layers, demonstrating that cell features learned by stACN are discriminative for the complicated structure of spatial domains. To fully investigate the capacity of various algorithms for learning features, we perform distribution density estimation for Layer1 and Layer2 with cell features learned by each approach. Distributions of features learned by stACN for Layer1 and Layer2 are significantly different (Fig 2 **E** left panel, p=0.025, Kolmogorov-Smirnov Test), whereas those obtained by baselines are non-significant except for stLearn (Fig 2 **E** right panel, SEDR: p=0.23, Kolmogorov-Smirnov Test, Fig E in S1 Text). These results further indicate that compatible features of cells are more precise in characterizing spatial domains. We further exploit relations of spatial domains obtained by various algorithms with UMAP and PAGA (the partition-based graph abstraction of spatial domains) [50], which is shown in Fig 2 **F** and Fig E in S1 Text. Specifically, various cortical layers identified by stACN are well organized from Layer1 to Layer6 and WM, whereas domains obtained by baselines fail to discriminate these layers. Specifically, stLearn fails to distinguish WM and cortex layers, SEDR mixes Layer1, Layer6, and cortical layer, SpaGCN cannot separate Layer1, Layer2, and Layer3, and STAGATE mixes Layer4 and Layer5. These results demonstrate that compatible features of cells are more precise in representing spatial domains in the DLPFC dataset.

Then, we further evaluate the performance of stACN with mouse brain SRT data by comparing the identified spatial domains with the brain anatomical reference annotations of Allen Mouse Brain Atlas (Fig 2 **G** left panel). stACN accurately identifies the cerebellar cortex and dentate gyrus within the hippocampal region of the mouse brain, along with the cornu ammonis 1(Type 16), cornu ammonis 3(Type 17), and dorsal gyrus(Type 18) in the coronal section, all of which align well with the manual annotations available at https://atlas.brain-map.org/ (Fig 2 **H**). Quantitative evaluation using Silhouette Coefficient (SC) and Davies–Bouldin (DB) scores indicates that the spatial domains identified by stACN exhibit strong compactness and separation, comparable to STAGATE (Fig E in S1 Text). In addition, distribution density estimation of cell features is also executed, where features of cells of stACN are significantly different among these three regions (Fig 2 **I**, p = 2.2E-5: Type 16 vs. Type 17, p = 4.0E-11: Type 16 vs. Type 18, Kolmogorov-Smirnov test).

### stACN improves interpretability of spatial domains with connection to topology of cell networks

stACN jointly learns cell features by integrating cell spatial and expression networks to construct an integrated one (affinity graph of cells), where the structure of spatial domains is automatically reflected by the topological structure of the graph. One of the major motivations of stACN is to improve the interpretability of spatial domains with cell networks. Thus, it is natural to investigate the association between spatial domains and the topological structure of the affinity graph of cells.

**Fig 3:**
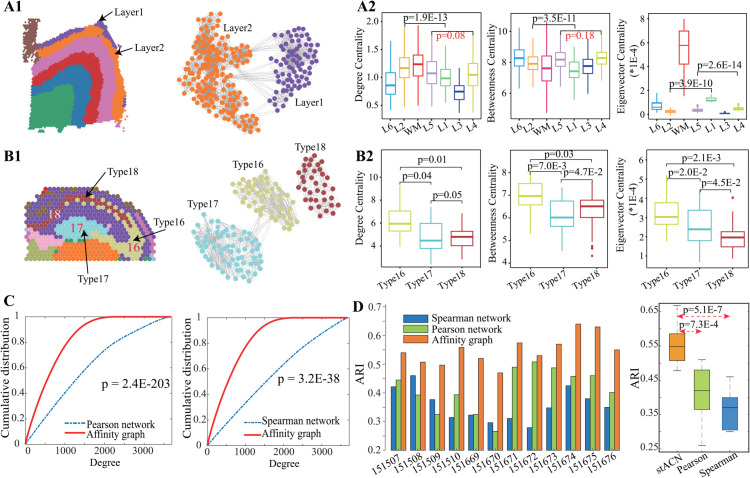
stACN improves interpretability of features by connecting with topology structure of cell networks. **(A1)** Visualization of spatial domains identified by stACN for slide 151675 in DLPFC dataset (left), and topological structure of subnetworks induced by Layer1 and Layer2 from affinity graph of cells learned by stACN (right). **(A2)** Distributions of topological indexes of cells within various spatial domains identified by stACN, including degree (left), betweenness (middle) and eigenvector centrality (right). **(B1)** Visualization of the cornu ammonis1(CA1, Type 16), cornu ammonis3(CA3, Type 17) and dorsal gyrus(Type 18) domain identified by stACN in mouse brain, and topological structure of subnetworks induced by Type 16, 17 and 18 from cell affinity graph learned by stACN. **(B2)** Distributions of topological indexes of cells within various spatial domains identified by stACN, including degree (left), betweenness (middle) and eigenvector centrality (right). **(C)** Cumulative distributions of degree for the cell affinity graph learned by stACN and cell expression network with Pearson coefficient (left) and Spearman (right) for slide 151675 in DLPFC, where x-axis denotes degree and Kolmogorov-Smirnov test is for significance. **(D)** ARIs of stACN with various types of networks, including cell affinity graph, Spearman, and Pearson networks, for each slide in DLPFC data (left). Distributions of ARIs of stACN for DLPFC data (right).

Fig 3 **A1** left panel shows that stACN precisely identifies Layer1 and Layer2 in slide 151675 of the DLPFC dataset, and we further investigate the relationship between Layer1/2 and the cell affinity graph, where sub-networks induced by Layer1/2 are visualized in Fig 3 **A1** (right). Surprisingly, these layers correspond to two graph clusters, i.e., connectivity of cells within each cluster is strong, and weak across clusters. These results demonstrate that stACN successfully transforms spatial domains into graph clusters in the cell affinity graph by manipulating compatible cell features. Furthermore, we analyze the relationship between all spatial domains and the affinity graph with various topological indices, such as degree (the sum of the weights of edges connected to a cell), betweenness (the number of paths across a cell), and eigenvector centrality (the importance of cells derived from the eigenvector of the adjacency matrix). Fig 3 **A2** depicts the distributions of cells in various domains in terms of degree (left), betweenness (middle), and centrality (right), respectively. It is obvious that cells in various domains significantly differ, implying that the topological structure of networks precisely characterizes the structure of spatial domains. For example, the average degree centrality is 0.91, 1.18, 1.17, 1.09, 1.02, 0.73, and 1.07 for L6, L2, WM, L5, L1, L3, and L4, respectively (p = 1.9E-13: L1 vs. L2, p = 1.7E-51: L6 vs. L2, p = 7.6E-58: L6 vs. WM, Student’s t-test). These results account for why stACN is superior to current baselines because it fully exploits the structure of cell networks, providing additional complementary information to the features of cells. These associations result in two subsequent advantages. First, stACN directly improves the interpretability of spatial domains because relations of cells are explicitly described, where the boundary and structure of domains are associated with the topological structure of networks. Second, stACN also provides an alternative to model spatial domains by exploiting indirect topological relations.

Then, we check whether the association between spatial domains and cell networks is sensitive to various datasets. Analogously, we conduct the same association analysis on the mouse brain dataset, where the cornu ammonis1(CA1, Type 16), cornu ammonis3(CA3, Type 17), and dorsal gyrus(Type 18) domains are accurately predicted by stACN (Fig 3 **B1** left). The topological structure of these three spatial domains also corresponds to modules in the cell affinity graph learned by stACN (Fig 3 **B1** right). Consistently, the distributions of topological indexes of cells, such as degree(left), betweenness(middle), and centrality(right), of these domains also significantly differ (Fig 3 **B2**, p<0.05, Student’s t-test). These results further indicate that stACN is robust and precise in capturing associations between spatial domains and the topology of cell networks for various SRT data.

Furthermore, current baselines construct cell networks by taking spatial distances as backbone and calculate similarity of cells in terms of expression profiles of cells with Pearson or Spearman coefficient, whereas stACN learns an affinity graph of cells with compatible features of cells. Thus, we investigate differences among cell networks generated with various algorithms, and find that distributions of node degree for these networks are significantly different (p=2.4E-203: Pearson vs. affinity graph, p=3.2E-38: Spearman vs. affinity graph, Kolmogorov-Smirnov test, Fig 3 **C**). Then, we investigate how these cell networks affect the performance of spatial domains with the third-party Leiden clustering [12], and find that algorithms are much more precise on the network learned by stACN than others for all slices in the DLPFC dataset (Fig 3 **D**). Specifically, average ARI is 0.414, 0.363 and 0.555 for network with Pearson, network with Spearman, and affinity graph for DLPFC dataset (p=7.3E-4: Pearson vs. affinity, p=5.1E-7: Spearman vs. affinity graph, Student’s t-test), further proving that stACN precisely captures intrinsic structure of spatial domains by associating with topology of cell networks, thereby improving interpretability of cell features.

### stACN accurately identifies of cancer and non-cancer spatial domains

**Fig 4:**
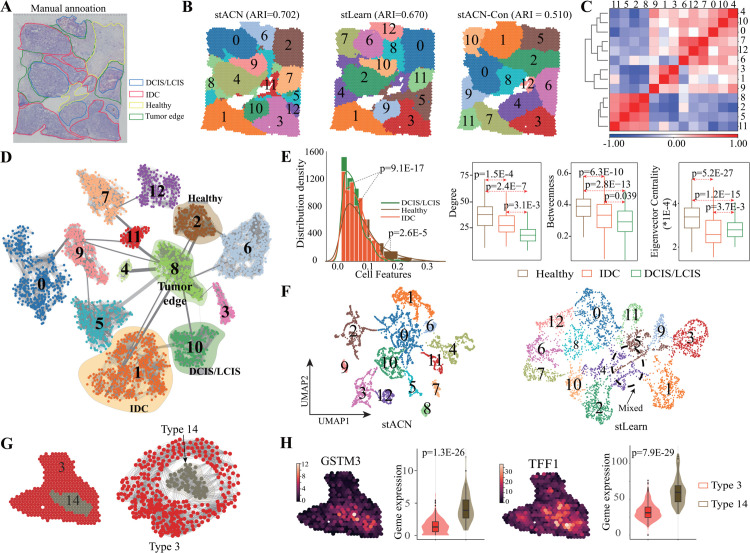
stACN accurately identifies of cancer and non-cancer spatial domains. **(A)** Visium SRT data of breast cancer annotated by pathologists consists of IDC (invasive ductal carcinoma), DCIS (ductal carcinoma in situ), LCIS (lobular carcinoma in situ), tumor edge, and healthy region. **(B)** Spatial domains identified by stACN (left), stLearn (middle), and stACN-Con (right). **(C)** Heatmap of Pearson correlation coefficient among domains (domain=13). **(D)** Visualization of topological structure of spatial domains for breast cancer data in cell affinity graph learned by stACN, where thickness of edges is proportional to edge weights. **(E)** Distribution density estimation of cells in IDC, DCIS/LCIS and Healthy domain in terms of the learned cell features, where x-axis denotes cell features, and Kolmogorov-Smirnov test is for significance (left), and Distributions of degree, betweennessand eigenvector of cells in IDC, DCIS/LCIS and Healthy domains identified by stACN (right), where p-value is calculated with Student’s t-test. **(F)** UMAP visualization of spatial domains identified by stACN (left) and stLearn (right), where dashed circle denotes mixed domains. **(G)** Hierarchical structure of domain 3 and 14 in SRT data (left), and topological structure of subnetwork induced by domain 3 and 14 in cell affinity graph (right). **(H)** Spatial distribution of expression of GSTM3 and TFF1 with regional annotation (left), and Violin plots of gene expression (right).

To investigate the generalization power of stACN for cancer spatial domain identification, the public human breast cancer SRT dataset (10 × Visium) with 3,798 spots and 36,601 genes is adopted (Table A in S1 Text). Fig 4 **A** visualizes H&E image of breast cancer tissue, where four typical morphotypes, such as ductal carcinoma in situ/lobular carcinoma in situ (DCIS/LCIS), healthy tissue (Healthy), invasive ductal carcinoma (IDC), and tumor surrounding regions with low features of malignancy (Tumor edge), are manually annotated by pathologists.

Parameter analysis shows that setting the number of clusters to 13 avoids over-segmenting these four manually annotated regions, which is consistent with DeepST [33] (Section D and Fig F in S1 Text). Performance of various algorithms for breast cancer spatial domain identification is shown in Fig 4 **B**, where spatial domains detected by stACN are highly consistent with the manual annotations, while less regional continuity and more outliers exist in domains identified by baselines (Fig F in S1 Text). In detail, ARI of stACN is 0.702, whereas that of stLearn and stACN-Con is 0.670 and 0.510, respectively. In other words, stACN is also applicable for cancer data, demonstrating that compatible cell features are precise for characterizing cancer spatial domains. To further investigate the spatial heterogeneity within the tumor, Fig 4 **C** visualizes heatmap of Pearson correlation coefficient of domains with expression profiles, which are clearly divided into normal (domain 2, 5, 8 and 11), and cancer group (the rest ones) group, demonstrating that tumor heterogeneity can also be reflected by spatial domains.

To check the association between cancer spatial domains and the cell affinity graph, the topological structure of spatial domains is illustrated in Fig 4 **D**, where the thickness of edges is proportional to edge weights. Surprisingly, all these domains are well reflected in the cell affinity network, where each domain corresponds to a cluster. These four typical domains, such as IDC, DCIS/LCIS, Tumor edge, and Healthy region, are well separated. Furthermore, the tumor edge region serves as the hub node to connect other domains, which may provide pathologists with potential clues for the diagnosis and therapy of breast cancer. To investigate how the learned cell features discriminate these four domains, Fig 4 **E** (left) depicts the distribution of cell features learned by stACN, where all these regions are significantly discriminated (p = 9.1E-17: DCIS/LCIS vs. Healthy; p = 2.6E-5: IDC vs. Healthy, Kolmogorov-Smirnov test). Surprisingly, MNMST significantly discriminates IDC and Healthy domains, and SEDR discriminates DCIS/LCIS and Healthy domains. But, all these baselines cannot discriminate these three domains (Fig G in S1 Text), indicating that stACN improves the discriminative of features.

stACN captures differences among various domains because indirect relations are exploited, and Fig 4 **E** depicts topological indexes of cells in Healthy, Tumor edge, IDC, and DCIS/LCIS domains in terms of degree, betweenness, and eigenvector centrality that differ greatly (degree: p = 1.5E-4, Healthy vs. IDC; p = 2.4E-7, Healthy vs. DCIS/LCIS, Student’s t-test). Fig 4 **F** visualizes domains identified by stACN (left) and stLearn (right) with UMAP, where domains identified by stACN are well separated, and these by other algorithms are mixed, particularly, these domains within the dashed circle (Fig G in S1 Text). These results further demonstrate that stACN is also precise in characterizing heterogeneous cancer spatial domains. To analyze genes within spatial domains, 178 differentially expressed genes (DEGs) (|*log*_2_ (fold change)|≥2; adjusted p<0.05) between domain IDC and DCIS/LCIS are obtained (Fig H in S1 Text, S1 Table). Functional enrichment analysis is executed to obtain the biological functions of DEGs (Fig H in S1 Text, S2 Table), where up-regulated genes are associated with immune-related pathways (p = 1.7E-2, Hypergeometric test), and down-regulated ones correspond to signal pathways of fibroblast migration (p = 2.8E-2, Hypergeometric test). These DEGs, such as COX6C and CCND1, are highly related to therapy [51] and metastasis [52] of breast cancer (Fig H in S1 Text). Furthermore, APOE, HEBP1, APOC1, CD24, AQP3, and NUPR1 serve as biomarkers for the infiltration of tumor-associated macrophages (TAM) [53–55].

Cancer spatial domains can be further divided into smaller sub-domains, reflecting a hierarchical structure that arises from high heterogeneity within the tumor. By setting the number of domains to 20, stACN captures intra-tumor heterogeneity at a finer resolution. Specifically, the IDC domain [33,56] can be further divided into two sub-domains (domains 3 and 14, Fig 4 **G** (left)), reflecting biologically meaningful intra-tumor heterogeneity. Strikingly, heterogeneity of cancer spatial domains is also reflected with topological structure of cells as shown in Fig 4 **G** (right), where distributions of topological indexes and features of cells between domain 3 and 14 domains also differ greatly, showing that cell networks are effective for characterizing complicated cancer spatial domains. Analogously, the comparison between domains 3 and 14 is performed, where a similar tendency repeats (Fig I in S1 Text). Fig 4 **H** illustrates differentially expressed gene GSTM3 and TFF1 between domains 3 and 14, where the spatial distribution of gene expression of these two genes indicates they are up-regulated in domain 14, and gene expression is significant between these two domains (p = 1.3E-26 for GSTM3, p = 7.9E-29 for TFF1). Evidence shows that GSTM3 is a multi-drug resistance gene [57], and TFF1 is associated with tumor differentiation [58].

Evidence shows that biomarker genes are associated with survival time of patients [59], and we hypothesize that DEGs associated with cancer spatial domains can also predict survival time of patients. Based on gene expression profiles and clinical information from various cancer types in The Cancer Genome Atlas (TCGA, https://www.cancer.gov), we found that the differentially expressed genes (DEGs) between the IDC and DCIS/LCIS domains are significantly associated with patient survival time, as revealed by Kaplan–Meier survival analysis. Specifically, 17.4% DEGs separate patients into high- and low-risk groups with significant survival time (Fig I in S1 Text, p<0.05).

### stACN effectively performs graph denoising for SRT data

Great evidence proves the existence of noise in SRT data, largely due to delicate procedures to preserve transcriptional and spatial information, posing a great challenge in removing noise. Moreover, extensive experiments demonstrate that denoising significantly enhances the performance of SRT data analysis [39,40]. Different from current stand-alone algorithms, such as Sprod [41], DIST [60], and MIST [42], stACN adopts a graph denoising strategy, where noise is characterized and removed by exploiting the topological structure of cell networks. Specifically, stACN learns the cell expression network by modeling attributes of cells with self-representation learning, and jointly removing noise on edges of cell spatial and expression networks using a low-rank constraint (Section Methods).

We first investigate performance of stACN for denoising by comparing with the typical stand-alone algorithms, such as Sprod, MIST and DIST, with the simulated data [61], where two factors are involved, i.e., the number of clusters and noise level (Table B in S1 Text). stACN achieves the best performance as the number of clusters increases from 6 to 15 (Fig J in S1 Text), demonstrating that stACN is insensitive to perturbation of the number of clusters. Then, we validate the performance of different approaches for denoising on simulated data with various noise levels. Performance of all these baselines dramatically decreases as the level of noise increases, whereas stACN is much more stable and precise than baselines (Fig J in S1 Text). Furthermore, visualization of clusters obtained by various algorithms from the noised simulated data demonstrates that clusters identified by stACN are more compact and well separated from each other (Fig J in S1 Text), indicating the quality of clusters is high. These results show that stACN is more precise and robust for noised simulated data than these stand-alone algorithms for denoising, demonstrating that graph denoising is promising for characterizing and removing noise.

**Fig 5:**
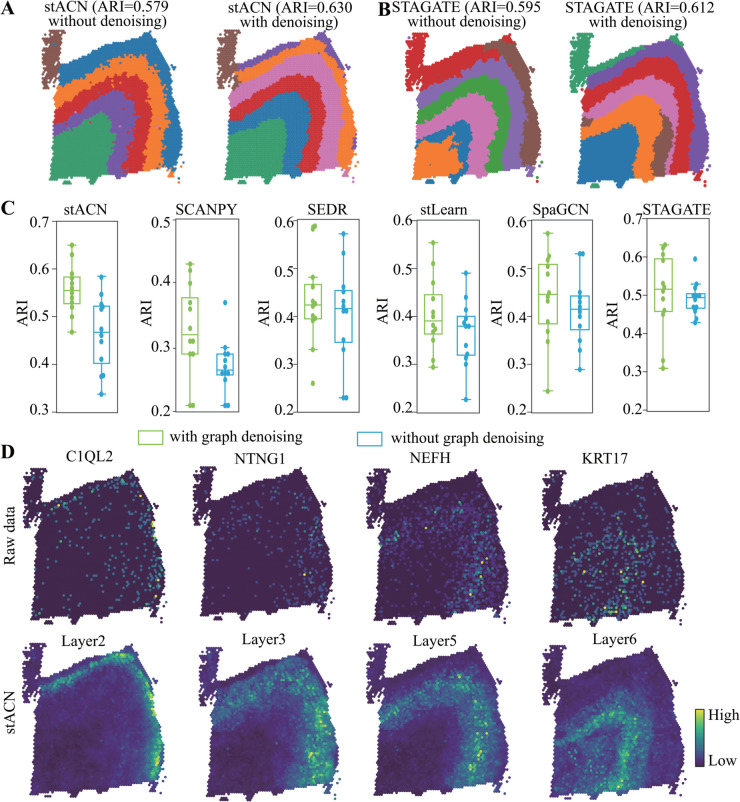
stACN effectively removes noise in SRT data with graph denoising strategy. **(A)** Visualization of spatial domains identified by stACN for slide 151675 in DLPFC data without denoising (left) and with denoising (right), respectively. **(B)** Performance of STAGATE for spatial domains for slide 151675 in DLPFC data (left), and performance of STAGATE with graph denoising provided by stACN for the same slide (right). **(C)** Distributions of ARIs of various algorithms with and without graph denoising of stACN for identifying spatial domains in DLPFC data. **(D)** Visualizations of the original (up) and denoised SRT data (bottom) for layer-marker genes in for slide 151675, where each column corresponds to one layer, respectively.

To validate the contribution of graph denoising, we first compare the performance of stACN with and without denoising on slide 151675 of the DLPFC dataset, where spatial domains are visualized in Fig 5 **A**. Graph denoising substantially enhances the performance of algorithms, where the ARI of stACN without denoising is 0.579, which increases to 0.630 with denoising. Specifically, Layer1 and Layer2 are well discriminated after graph denoising, which are mixed without denoising. These results demonstrate that removing noise in SRT data is crucial, which is consistent with the assertion in Refs. [39,40]. To examine whether graph denoising acts synergistically with stACN, we further test several graph-based baselines for spatial domain identification using both the original and denoised data(i.e., the affinity cell network output by stACN). Strikingly, STAGATE exhibits a remarkable improvement when applied to the denoised data (Fig 5 **B**), where ARI of STAGATE with the original data is 0.595 for slide 151675, and it increases to 0.612 with graph denoising. Furthermore, the performance of all these baselines improves on slide 151675 in the DLPFC dataset(Fig K in S1 Text), demonstrating that stACN precisely characterizes and removes noise in data.

To further investigate whether graph denoising is sensitive to slice 151675, we repeat the procedure for all slices in the DLPFC dataset, and the distribution of ARI of various algorithms is shown in Fig 5 **C**. Notably, all these algorithms exhibit an improvement in performance with the clean data denoised by stACN. In detail, ARI with graph denoising of stACN is 0.559 ± 0.049, whereas it is 0.320 ± 0.041 for SCANPY, 0.423 ± 0.088 for SEDR, 0.397 ± 0.073 for stLearn, 0.445 ± 0.087 for SpaGCN, and 0.535 ± 0.099 for STAGATE, respectively. These results show that stACN provides an effective and efficient graph denoising strategy for SRT data. Moreover, we validate the performance of graph denoising with breast cancer SRT data, where ARI of stACN without denoising decreases from 0.702 to 0.660 (Fig K in S1 Text), indicating that noise dramatically masks the structure of cancer spatial domains. Furthermore, performance of all these baselines also improves with graph denoising of stACN for cancer SRT data (Fig K in S1 Text), demonstrating stACN precisely captures and removes noise of cancer SRT data.

To further check the quality of graph denoising, we visualize the layer-specific biomarker genes (differentially expressed genes) for each layer with the original and denoised expression profile of cells as shown in Fig 5 **D**, where C1QL2 (Layer2), NTNG1 (Layer3), NEFH (Layer5), and KRT17 (Layer6) are illustrated. It is obviously observed that graph denoising significantly improves the structure of various layers, i.e., the laminar enrichment of these layer-marker genes is clearly augmented, facilitating the identification of spatial domains. Except for DLPFC data, graph denoising of stACN also augments the structure of spatial domains in human breast cancer data (Fig K in S1 Text). These results demonstrate that stACN effectively leverages the topological structure of cell networks to remove noise, thereby providing an alternative for removing noise in SRT data.

Since stACN joins graph denoising and spatial domain identification, it is natural to check whether the joint denoising strategy is superior to the separating strategy. In detail, the separating strategy first performs denoising with stand-alone algorithms and then identifies spatial domains on the denoised data. Fig L in S1 Text visualizes spatial domains identified by various algorithms on the raw and denoised data with different approaches, where each column denotes an algorithm, and each row corresponds to a visualization of spatial domains identified by various algorithms for either the raw or denoised data. It is easy to assert that all these algorithms, except for SCANPY, increase performance on the breast cancer data denoised by stACN, whereas data denoised by stand-alone approaches result in a decrease in performance of the algorithms. These results further demonstrate that the joint strategy of stACN is more precise than the separating strategy with stand-alone denoising algorithms.

stACN simultaneously employs data augmentation, graph denoising, and feature learning, and we investigate the role of each component with an ablation analysis. Performance of variants of stACN, including stACN without data augmentation and graph denoising, without data augmentation, and without graph denoisng, on various datasets, which is shown in the Fig M in S1 Text, where panel **A** is for the DLPFC dataset and **B** for the breast cancer dataset. From these panels, we assert that data augmentation and graph denoising are critical for stACN since both of them improve the performance of stACN. In detail, graph denoising results in 5.1% (4.2%) improvement for DLPFC (Breast cancer), whereas data augmentation brings in 6.7% (6.0%) improvement for DLPFC (Breast cancer), respectively. These results demonstrate that data augmentation and graph denoising improve the performance of stACN, which are critical components of stACN. And, the quality of features learned by stACN dominates all components.

Except for the affinity graph of cells, we also investigate whether the clean graphs learned by stACN also remove noise in SRT data. Performance of SpaGCN and MNMST for the raw and clean graphs learned by stACN for slice 151675 of the DLPFC dataset is investigated, where the raw graphs are directly constructed from spatial transcriptomics data. Results show that ARIs of these two algorithms also improve (Fig N in S1 Text), indicating that stACN removes noise from the data at multiple stages. Performance of these algorithms on all slices of the DLPFC dataset further demonstrates that clean graphs learned by stACN also enhance the accuracy of spatial domain identification (Fig N in S1 Text). Analogously, performance of SpaGCN and MNMST on the breast cancer dataset is consistent with that of the DLPFC dataset (Fig N in S1 Text), showing that the clean graphs learned by stACN also denoise SRT data to some extent.

### stACN enhances integrative analysis of SRT data

The accumulated SRT data pose a great challenge to the integration of them, and stACN provides an alternative for addressing this issue from two perspectives, i.e., integrating multiple slices of specific tissues, and removing batch effect in various datasets.

**Fig 6:**
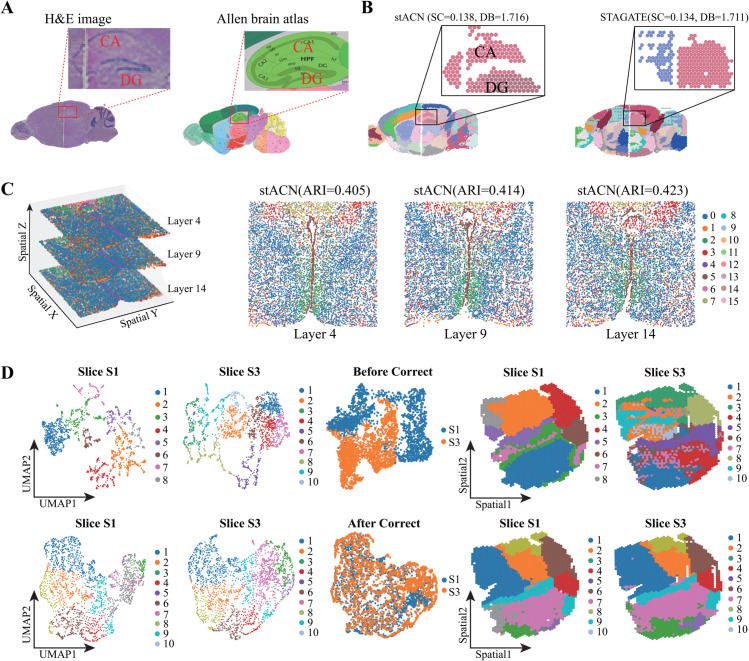
stACN enhances integrative analysis of SRT data. **(A)** H&E images of mouse anterior and posterior brain datasets of 10 × Visium, which are horizontally aligned (left). The zoomed in region consists of cornu ammonis(CA) and dentate gyrus(DG) domain. The corresponding anatomical Allen Mouse Brain Atlas (right). **(B)** Spatial domains identified by stACN (left) and STAGATE (right), where CA and DG across different slices. **(C)** 3D coordinates of MERFISH data for mouse hypothalamic preoptic region with slice 4, 9, and 14 (left), and spatial domains identified by stACN for each slice (right). **(D)** Visualization of SRT data for mouse breast cancer, where slice S1 and S3 are from different batches (first two columns), visualization of slice S1 and S3 with and without removing batch effect (the third column), and spatial domains identified by stACN with and without removing batch effect (last two columns), respectively.

Large tissues require an integrative analysis of multiple slices to cover the whole section. For example, Visium SRT data for mouse brain cover anterior and posterior brain slices is shown in Fig 6 **A** (left), where the zoomed region contains CA(cornu ammonis) and DG(dentate gyrus) across the left and right slices. PASTE [62] is employed to horizontally align these two slices into an aligned one, and annotation of domains for the aligned slice is from Allen Mouse Brain Atlas (Fig 6 **A** right). And, stACN not only precisely identifies CA and DG regions, but also identifies these domains across different slices, whereas STAGATE fails to identify domains across various slices (Fig 6 **B**). Careful comparison of these two panels in Fig 6 **B** demonstrates that, although STAGATE and stACN have similar SC and DB scores, stACN accurately preserves the structure of domains in different slices, implying that the attributed network-based model is applicable for integrating horizontally aligned SRT data. Analogously, sub-networks induced by CA and DG from the in-cell affinity graph obtained by stACN demonstrate that these two domains are clearly separated as clusters of cells, implying that stACN precisely learns consistent features of cells across various slices. In detail, cells in the CA and DG domains significantly differ in terms of various topological indexes and distribution of features (Fig O in S1 Text).

Except for a horizontally aligned slice, stACN is also applicable to integrate adjacent slices of the same tissue, i.e., it effectively integrates multiple vertically adjacent slices. stACN provides two different manners for the vertical integration of SRT data, i.e., data fusion and software fusion, where the former strategy augments each slice with adjacent ones, and then performs spatial domain identification with the augmented slice. And, the latter strategy takes the fused slice of omic SRT data produced by other approaches, such as PASTE [62], as input. Fig 6 **C** (left) visualizes 3D spatial coordinates of MERFISH data for the mouse hypothalamic preoptic region with slices 4, 9, and 14 [63], and spatial domains identified by stACN for each slice (right). It is obvious that stACN accurately identifies spatial domains for each slice with ARI is greater than 0.4 (Fig 6 **C** right), which is significantly higher than baselines (Fig O in S1 Text). These results prove that stACN is more precise in characterizing complicated spatial domains since it reaches a good balance among adjacent slices.

Then, we further validate the performance of stACN for vertical integration of the DLPFC dataset with four slices, i.e., 151673, 151674, 151675, and 151676. Experimental results show that integrative analysis of multiple slices promotes accuracy of algorithms (Fig O in S1 Text). In detail, the ARI of the integrative analysis is 0.602, whereas it is 0.550 without integrating adjacent slices. Moreover, we validate the performance of stACN for integrating SRT data in a second manner, where PASTE integrates four slides into a center slide. And, stACN precisely detects spatial domains in the aligned center slide with ARI 0.650, whereas it is 0.490 for STAGATE (Fig P in S1 Text). It implies that stACN precisely characterizes the structure of spatial domains from data provided by the third party, demonstrating its superiority for integrative analysis of SRT data.

Moreover, the stACN also effectively removes batch effect from spatially resolved omic data, which is also investigated. The typical methods, including STAGATE and SEDR, address this issue with additional modules, while stACN directly handles it. In detail, stACN first employs SCANPY [44] to stack multiple slices from various batches, and then directly identifies spatial domains from the stacked slice as input. It learns the compatible cell features from the constructed network with tensor singular value decomposition, where consistent features of cells across different slices or batches are learned, thereby removing batch effect in feature space. Fig 6 **D** (left) visualizes two slides of mouse breast cancer SRT data from two different batches, where the batch effect is severe since domains in S1 and S3 are inconsistent [38]. Fig 6 **D** (right) visualizes spatial domains identified by stACN for slices without (above) and with (bottom) removing batch effect, where spatial domains of different slides are highly consistent after removing batch effect, demonstrating that stACN is also effective for removing batch effect. Moreover, stACN outperforms baselines on removing batch effect in slices 151673, 151674, 151675, and 151676 in the DLPFC dataset (Fig P in S1 Text). Furthermore, stACN is also precise in removing noise from vertically stacked slices. Therefore, stACN effectively removes batch effect in vertically aligned SRT slices, proving that the attributed network-based model is also promising for removing batch effect.

### stACN is applicable for spatial omics data with various platforms

Various technologies have been developed for generating SRT data, and it is necessary to validate the applicability of stACN. Therefore, two additional types of data, including mFISH (imaging-based molecular data) [64,65] and Stereo-seq (high-resolution spatial transcriptomics data) [66], are selected (Table A in S1 Text).

**Fig 7:**
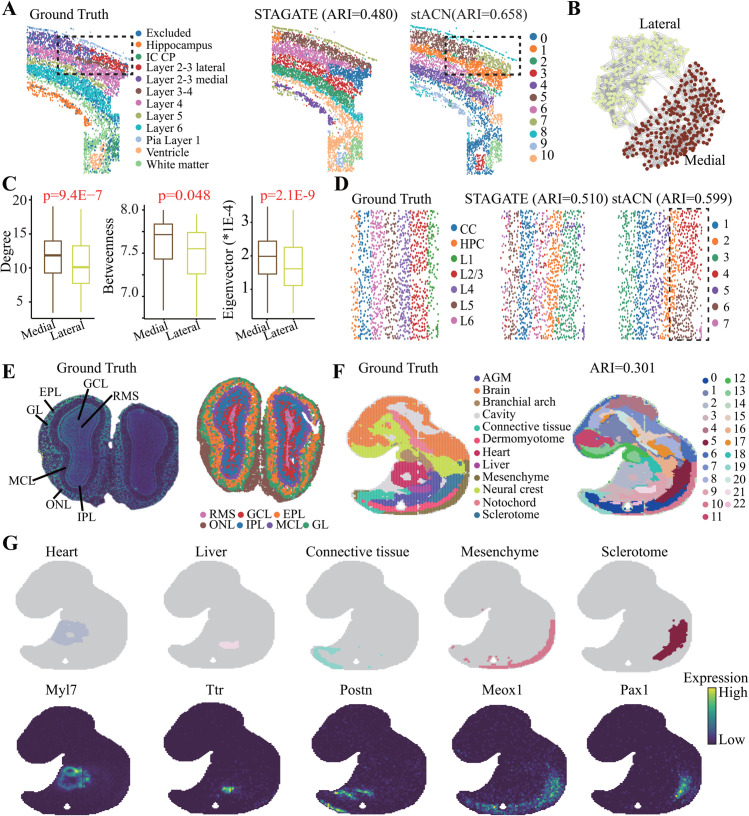
stACN is insensitive to platforms. **(A)** Ground truth of regional annotations of osmFISH SRT data (left), spatial domains identified by STAGATE (middle), and by stACN (right). **(B)** Topological structure of subnetwork induced by the Lateral and Medial domain. **(C)** Distribution of degrees (left), betweenness (middle) and eigenvector (right) of cells in the Lateral and Medial domain (Student’s t-test). **(D)** Ground truth of layer structure of STARmap data (left), spatial domains detected by STAGATE (middle), and stACN (right). **(E)** Visualization of SRT data generated with Stereo-seq platform (left), and spatial domains identified by stACN (right). **(F)** Tissue domain annotations of the E9.5 mouse embryo data generated with Stereo-seq platform, and spatial domains identified by stACN (right). **(G)** Visualization of spatial domains identified by stACN for each tissue and the corresponding marker gene expressions, where each column denotes a tissue.

The non-lattice-shaped spatial transcriptomics dataset generated by osmFISH [65] for the mouse brain somatosensory cortex with layer annotation is illustrated (Fig 7 **A** left), which contains 5,328 cells. And, stACN is superior to other baselines for spatial domains in osmFISH dataset (Fig 7 **A**, Fig Q in S1 Text). Specifically, stACN precisely distinguishes lateral and medial regions that are mixed by other baselines (Fig 7 **A** right). Sub-networks of the lateral and medial domain demonstrate that these regions can be precisely characterized with a cell affinity graph (Fig 7 **B**), and cells of various domains significantly differ in terms of topological indexes (Fig 7 **C**, Degree: 11.5±0.7 (Lateral) vs.7.3±1.0 (Medial), p=9.4E-7, Student’s t-test). Furthermore, the distribution of learned cell features significantly differs between the two domains (Fig Q in S1 Text), indicating that stACN improves the discriminative power of features. These results demonstrate that stACN is also applicable to osmFISH data since it precisely characterizes and obtains spatial domains.

The STARmap dataset [64] for the mouse visual cortex, containing 1,207 cells and seven layers, is also adopted (Fig 7 **D** left). Although stACN performs slightly worse than BASS, it outperforms all other methods(Fig 7 **D**). The reason is that BASS provides a better way to characterize the spatial domain in SRT data generated by image-based technologies. Notably, stACN successfully identifies the L1 and L2/3 domains, which are not distinguished by other methods(Fig 7 **D**). In detail, the differences between the L1 and L2/3 layers are also reflected in the topological structure of the cell affinity graph and the learned features (Fig Q in S1 Text). Furthermore, we evaluate the performance of stACN on SRT data generated by Stereo-seq for mouse olfactory bulb tissues [66], where stACN precisely identifies the spatial domains (Fig 7 **E**, right).

Finally, the Stereo-seq dataset [66] for mouse embryos at E9.5 is selected, which consists of 5,913 bins and 25,568 genes (Fig 7 **F** left). stACN precisely identifies spatial domains that match the annotated regions (Fig 7 **F** right). More importantly, each spatial domain is concordant with the known marker genes of different tissues. For example, the liver region is marked by Ttr, the mesenchyme by Meox1, the Sclerotome by Pax1, the heart by Myl7, and the connective tissue by Postn (Fig 7 **G**). These results demonstrate that stACN can accurately delineate tissue locations and capture the corresponding marker gene expression during embryonic development, providing insights into spatial tissue architecture and suggesting that the model is applicable across datasets generated with different platforms.

## Discussion and conclusion

Advances in next-generation sequencing ensure accurate transcription of genes at the cell level by preserving spatial locations, which provide a great opportunity for revealing mechanisms of biological systems. Mining the accumulated SRT data facilitates the understanding of the structure and function of tissues because cell populations execute their functions at various regions of organisms, which cannot be identified from data generated from the traditional bulk-based sequencing platforms.

However, analyzing SRT data is highly non-trivial because of the heterogeneity, extraordinary sparsity, and noise of data, which pose a great challenge in designing effective algorithms. In this study, we focus on identifying spatial domains in SRT data by proposing a novel attributed cell network-based model (aka stACN), where the expression and spatial location of cells are converted into an attributed graph. The proposed algorithm constructs cell spatial and expression networks with representation learning, removes noise in data with a graph model, performs compatible feature learning with joint matrix decomposition, and executes spatial domain identification by exploiting the structure of the cell affinity graph. What we want to point out is that the organization of stACN is flexible, which can be easily extended under various circumstances. First, stACN automatically learns cell networks from SRT data for analysis, and it can also take cell networks from third parties as inputs, thereby facilitating applications for various users with different backgrounds. Second, although stACN only exploits the expression and spatial information of SRT data, it serves as a flexible framework to integrate additional information, such as histological images, with a simple extension.

Extensive experiments demonstrate that stACN is an alternative for spatial domain identification in SRT data, where its superiority is summarized from several perspectives. First, stACN significantly improves compatibility of cell features by jointly factorizing the cell expression and spatial networks, which provides a better way to characterize and model the structure of spatial domains (Fig 2). Second, stACN improves the interpretability of spatial domains by connecting to the topological structure of cell networks (Fig 3). Third, stACN precisely identifies cancer spatial domains, which provides biologists with potential clues for further studies (Fig 4). Fourth, stACN provides an effective graph denoising strategy, which serves as the first and critical step for pre-processing SRT data (Fig 5). Finally, stACN also efficiently integrates omic SRT data with different platforms, thereby extensively extending its application (Figs 6 and 7).

Notice that there are ample opportunities to further enhance the performance of stACN, which will be addressed in further studies. First, stACN makes use of cell networks to represent and identify spatial domains in SRT data, where the computational complexity is high because graph pattern mining is time-consuming. Actually, the rapid development of SRT technologies generates a large amount of super-resolution SRT data. Thus, how to accelerate stACN for SRT data with millions of cells is promising for high-resolution SRT data. Second, the current framework assumes uniform weighting between spatial and expression information, which may not optimally reflect tissue-specific heterogeneity or microenvironmental influences. Thus, how to develop adaptive weighting schemes to balance the contributions of spatial and molecular information represents an important direction for improving the accuracy of spatial domain identification. Third, expression and spatial information are insufficient to fully characterize complicated spatial domains. Thus, how to combine SRT data, single-cell RNA-sequencing, and bulk-based data represents a promising direction for identifying biological patterns.

In conclusion, we introduce a novel algorithm to simultaneously remove noise and identify spatial domains with a joint learning strategy model, where noise is modeled with the downstream task. Experimental results demonstrate that it reveals complex tissue organization and discovers gene markers of spatial domains, providing an alternative for analyzing complex biological systems. Moreover, stACN demonstrates its superiority in identifying the spatial domain, integrating and denoising SRT data, and addressing various platforms. We also demonstrate the benefits of stACN through comprehensive analyses with various datasets. In all, stACN not only precisely identifies spatial domains, but also provides a novel graph denoising strategy for SRT data.

## Materials and methods

### Data preprocessing

Eight simulated and ten biological datasets (Table A in S1 Text) are employed to fully validate the performance of stACN. For all datasets, spots outside the main tissue regions are removed, and raw gene expression data are filtered, normalized, and log-transformed according to library size with SCANPY [44]. Seurat [45] is employed to remove unexpressed genes (expressed in fewer than 10 cells), and to select the top 3,000 highly variable genes (HVGs). BANKSY [37] is then used to augment the expression profiles of each cell. Dimensionality reduction is performed using principal component analysis (PCA), and the resulting low-dimensional embeddings serve as input for downstream analyses.

### Attributed cell network construction

Cell attributed graph G={V,A,X} consist of vertex set *V*, adjacent matrix $A\in R^{n\times n}$, and attributed matrix $X\in R^{n\times m}$, where vertices in *V* correspond to cells, adjacent matrix of *A* represents topological structure of *G* with element *a*_*ij*_ as weight on edge connecting the *i*-th and *j*-th cell, and attributed matrix *X* denotes expression profiles of *n* cells across *m* genes. Specifically, stACN constructs a spatial adjacency graph *A* using the K-nearest neighbor algorithm (following Squidpy [67]; the number of neighbors *k* is 5 for 10× Genomics, and 8 for imaging-based platforms; Section I and Fig S in S1 Text), where the edge weight *a*_*ij*_ between the *i*-th and *j*-th cell is inversely proportional to the Euclidean distance *r*_*ij*_ calculated from the spatial coordinates, i.e., $a_{ij}=r_{ij}^{-1}$.

### Mathematical model of stACN

Since stACN integrates graph denoising, compatible feature learning, and spatial domain identification (structural constraint), the mathematical model of stACN also consists of three major components.

On the graph denoising issue, stACN first learns a cell expression network with self-representation learning [68] by assuming that each cell can be effectively represented with its adjacent neighbors. Specifically, given attribute $X\in R^{n\times m}$, the *t*-th cell is represented as

$$x_{t}=g_{1}^{\left[e\right]}x_{1}+\cdots +g_{n}^{\left[e\right]}x_{n}$$ (1)

where $g_{i}^{\left[e\right]}$ and $x_{i}$ are the weight and attribute profile of the *i*-th cell, respectively. By jointly learning all cells, stACN learns the cell expression network *G*^[*e*]^ by minimizing the reconstruction error

$$𝒪(X,G^{\left[e\right]})=\|X-XW^{\left[e\right]}{\|}^{2},\;s.t.\;diag(W^{\left[e\right]})=0,W^{\left[e\right]}=(W^{\left[e\right]}{)}^{\prime },$$ (2)

where constraint ensures non-trivial solutions, i.e., no loop is allowed in network *G*^[*e*]^, $W^{\left[e\right]}\in R^{n\times n}$ is the adjacent matrix of *G*^[*e*]^, and $(W^{\left[e\right]}{)}^{\prime }$ is the transpose of matrix *W*^[*e*]^. According to Ref. [69], Eq 2 can be approximately solved by minimizing rank of $\left\|W^{\left[e\right]}\right\|$ as

$$𝒪(X,G^{\left[e\right]})=\|W^{\left[e\right]}{\|}_{*},\;s.t.\;diag(W^{\left[e\right]})=0,W^{\left[e\right]}=(W^{\left[e\right]}{)}^{\prime },$$ (3)

where $\|W^{\left[e\right]}{\|}_{*}$ denotes nuclear norm of $\left\|W^{\left[e\right]}\right\|$.

Since SRT data are full of noise, we assume noise is subject to the additive model, i.e.,

$$X=XW^{\left[e\right]}+E,$$ (4)

where $E\in R^{n\times m}$ denotes noise of $X\in R^{n\times m}$. We expect noise is sparse, and Eq 3 is re-written as

$$𝒪(X,G^{\left[e\right]})=\|W^{\left[e\right]}{\|}_{*}+\lambda \|E{\|}_{2,1},$$ (5)

$$s.t.X=XW^{\left[e\right]}+E,diag(W^{\left[e\right]})=0,W^{\left[e\right]}=(W^{\left[e\right]}{)}^{\prime },$$

where $\|E{\|}_{2,1}$ denotes the *l*_2,1_-norm constraint to ensure sparsity of *G*^[*e*]^. Furthermore, stACN extracts the principle features from *X* with linear projection $P^{\left[e\right]}\in R^{n\times n}$, which is formulated as

$$P^{\left[e\right]}X=P^{\left[e\right]}XW^{\left[e\right]}+E^{\left[e\right]}.$$ (6)

Analogously, stACN also perform denoising for spatial network *G*^[*s*]^ as

$$P^{\left[s\right]}A=P^{\left[s\right]}AW^{\left[s\right]}+E^{\left[s\right]},$$ (7)

where $P^{\left[s\right]}\in R^{n\times n}$ and $E^{\left[s\right]}\in R^{n\times n}$ are the projection matrix and noise matrix, respectively.

By integrating graph denoising of spatial and expression information, the objective function of the network noise model is formulated as

$$𝒪(X,G,E,P)=\|W^{\left[s\right]}{\|}_{*}+\|W^{\left[e\right]}{\|}_{*}+\lambda (\|E^{\left[e\right]}{\|}_{2,1}+\|E^{\left[s\right]}{\|}_{2,1}),$$ (8)

$$s.t.P^{\left[e\right]}X=P^{\left[e\right]}XW^{\left[e\right]}+E^{\left[e\right]},W^{\left[e\right]}=(W^{\left[e\right]}{)}^{\prime },P^{\left[e\right]}(P^{\left[e\right]}{)}^{\prime }=I,$$

$$P^{\left[s\right]}A=P^{\left[s\right]}AW^{\left[s\right]}+E^{\left[s\right]},W^{\left[s\right]}=(W^{\left[s\right]}{)}^{\prime },P^{\left[s\right]}(P^{\left[s\right]}{)}^{\prime }=I,$$

where parameter *λ* controls importance of noise, *I* is an n×n identity matrix, and $W^{\left[s\right]}\in R^{n\times n}$ denotes adjacent matrix of *G*^[*s*]^, respectively.

On the compatible feature learning issue, we formulate the learned graphs *W*^[*e*]^ and *W*^[*s*]^ as a tensor $𝒲=[W^{\left[e\right]},W^{\left[s\right]}]\in R^{n\times n\times 2}$, and learn compatible cell features with tensor singular value decomposition (t-SVD) [70]. The objective function for this issue is formulated as

$$𝒲=𝒰𝒮(𝒱{)}^{\prime },$$ (9)

where $𝒰=[U^{\left[e\right]},U^{\left[s\right]}]\in R^{n\times d\times 2}$ is the concatenation of left eigenvectors of *W*^[*e*]^ and *W*^[*s*]^, and *d* is the number of features. Then, we also expect features obtained by t-SVD are also low-rank, which are fulfilled with Schatten *p*-norm as Ref. [71]

$$\|𝒲{\|}_{Ⓢ}=(\sigma_{k}(W^{\left[s\right]}{)}^{p}+\sigma_{k}(W^{\left[e\right]}{)}^{p}{)}^{\frac{1}{p}},$$ (10)

where 0≤p≤1, $\sigma_{k}(W^{\left[e\right]})$ is the *k*-th singular value of *W*^[*e*]^.

By combining Eqs 8 and 10, the ultimate objective function of stACN is formulated as

$$𝒪(X,Z,E,P)=\|𝒲{\|}_{Ⓢ}+\lambda \|E{\|}_{2,1},$$ (11)

$$s.t.P^{\left[e\right]}X=P^{\left[e\right]}XW^{\left[e\right]}+E^{\left[e\right]},W^{\left[e\right]}=(W^{\left[e\right]}{)}^{\prime },P^{\left[e\right]}(P^{\left[e\right]}{)}^{\prime }=I,$$

$$P^{\left[s\right]}A=P^{\left[s\right]}AW^{\left[s\right]}+E^{\left[s\right]},W^{\left[s\right]}=(W^{\left[s\right]}{)}^{\prime },P^{\left[s\right]}(P^{\left[s\right]}{)}^{\prime }=I,$$

$$𝒲=[W^{\left[e\right]},W^{\left[s\right]}],E=[E^{\left[e\right]};E^{\left[s\right]}],$$

where parameter *λ* controls importance of noise (*λ*=0.001, Section D and Fig A in S1 Text).

Eq 11 is solved with ADMM (the alternating direction method of multipliers) [72], where optimizing rules for variables are deduced (Section A in S1 Text). The algorithm analysis is also presented (Section B in S1 Text). Furthermore, the running time, space, as well as acceleration of stACN with hardware are also proposed (Section G and Fig R in S1 Text).

### Clustering and visualization

The Leiden algorithm [12] is selected to obtain spatial domains by performing graph clustering on the affinity graph learned by stACN. For datasets with a known number of clusters, stACN executes a grid search over the resolution parameter between 0.1 and 2.5 with a step size of 0.01 to reach the desired number of clusters. To ensure reproducibility, all experiments are performed with a fixed random seed, and the clustering results are confirmed to be stable across repeated runs. The uniform manifold approximation and projection (UMAP) algorithm is used for visualization [50].

### Spatial trajectory inference

PAGA [73] infers trajectory by exploiting a topology-preserving map of cells, whose input includes a single-cell graph and clustering assignment. PAGA generates a graph of clusters, where vertices correspond to clusters, and edges represent similarity among clusters. Here, PAGA takes a cell affinity graph and domain information as input to generate a trajectory of domains.

### Benchmarking

To evaluate performance of stACN, state-of-the-art methods, such as SCANPY [44], STAGATE [21], stLearn [20], BayesSpace [18], SpaGCN [23], SEDR [24], Giotto [19], BASS [47], SpaDo [48], MNMST [22], STMGCN [49], and BANKSY [37], are selected. All these methods are implemented with optimal parameter values(Section H in S1 Text).

The adjusted rand index (ARI) and normalized mutual information (NMI) [74] are selected to measure the accuracy of spatial domains if the ground truth is known; otherwise, machine learning criteria are selected. In detail, two clustering metrics, namely the Silhouette Coefficient (SC) and the Davies-Bouldin (DB) score, are employed. SC is calculated as the mean intra-cluster distance, while DB is defined as the average similarity between each cluster and its most similar counterpart. Both metrics are implemented in scikit-learn (https://scikit-learn.org).

### stACN removes batch effect of multiple slices

stACN utilizes the conserved cells across various slices to correct the spatial location of cells in each slice, forcing cells from various slices to be consistent, which effectively removes batch effect. In detail, stACN first stacks multiple slices from various batches with SCANPY and constructs an attributed cell network. Then, stACN learns the compatible cell features from the constructed network with tensor singular value decomposition, where consistent features of cells across different slices or batches are enhanced, thereby removing batch effect in feature space.

## Supporting information

S1 Text Supplementary materials. (DOCX)

S1 Table Differentially Expressed Genes (DEGs) Between IDC and DCIS/LCIS Spatial Domains in a Human Breast Cancer Dataset. (CSV)

S2 Table Gene Ontology Analysis of Differentially Expressed Genes Between IDC and DCIS/LCIS in a Human Breast Cancer Dataset. (CSV)

## Ethics approval and consent to participate

No ethical approval was required for this study. All utilized public datasets were generated by other organizations that obtained ethical approval.
