# Supplementary material for: Network models for bridging denoising and identifying spatial domains of spatially resolved transcriptomics
Source: PLoS Comput Biol. 2026 Jan 13;22(1):e1013867. doi: 10.1371/journal.pcbi.1013867 (PMC12799013; doi:10.1371/journal.pcbi.1013867)
Supplement: S1 Text — (DOCX) [file pcbi.1013867.s001.docx]

Supplementary Materials for

**Network models for bridging denoising and identifying spatial domains of spatially resolved transcriptomics**

Haiyue Wang, Wensheng Zhang, Zaiyi Liu, Xiaoke Ma**.*

*Corresponding author. Email: [xkma@xidian.edu.cn](mailto:xkma@xidian.edu.cn).

Supplementary Notes for stACN

(A) Optimization of stACN

By introducing the auxiliary tensor variable $\mathcal{Z}$, the objective function of stACN is equivalent to the following problem, i.e.,

$$min\left\| \mathcal{Z} \right\|_{Ⓢ}+\lambda\left\| E \right\|_{2,1},$$

$s.t. P^{\left[ e \right]}X=P^{\left[ e \right]}XW^{\left[ e \right]}+E^{\left[ e \right]},W^{\left[ e \right]}=\left( W^{\left[ e \right]} \right)^{'}, P^{\left[ e \right]}\left( P^{\left[ e \right]} \right)^{'}=I,$

$P^{\left[ s \right]}A=P^{\left[ s \right]}AW^{\left[ s \right]}+E^{\left[ s \right]},W^{\left[ s \right]}=\left( W^{\left[ s \right]} \right)^{'}, P^{\left[ s \right]}\left( P^{\left[ s \right]} \right)^{'}=I$ (1)

$$\mathcal{W=}\left[ W^{\left[ e \right]},W^{\left[ s \right]} \right],E=\left[ E^{\left[ e \right]};E^{\left[ s \right]} \right]\mathcal{,Z=W}$$

The augmented Lagrange function of Eq. (1) is formulated as:

$$\mathcal{\mathcal{L}}\left( \mathcal{Z,W,}E,P \right)=\left\| \mathcal{Z} \right\|_{Ⓢ}+\lambda\left\| E \right\|_{2,1}+\Phi\left( \mathcal{T}_{1}\mathcal{,W-Z} \right)+\Phi(\mathcal{T}_{2}, P^{\left[ e \right]}X-P^{\left[ e \right]}XW^{\left[ e \right]}-E^{\left[ e \right]})$$

$+ \Phi(\mathcal{T}_{3},P^{\left[ s \right]}A-P^{\left[ s \right]}AW^{\left[ s \right]}-E^{\left[ s \right]})$

$s.t. W^{\left[ e \right]}=\left( W^{\left[ e \right]} \right)^{'}, P^{\left[ e \right]}\left( P^{\left[ e \right]} \right)^{'}=I,W^{\left[ s \right]}=\left( W^{\left[ s \right]} \right)^{'}, P^{\left[ s \right]}\left( P^{\left[ s \right]} \right)^{'}=I$ (2)

$$\mathcal{W=}\left[ W^{\left[ e \right]},W^{\left[ s \right]} \right],E=\left[ E^{\left[ e \right]};E^{\left[ s \right]} \right]$$

where $\Phi\left( Y,C \right)=<Y,C>+\frac{\mu}{2}\left\| C \right\|^{2}$with $\left\langle\cdot,\cdot\right\rangle$denotes the inner product of matrices,$\mathcal{T}_{1}=[\mathcal{T}_{1}^{\left[ e \right]};\mathcal{T}_{1}^{[s]}]$,$\mathcal{T}_{2}$and $\mathcal{T}_{3}$ represent Lagrangian multipliers, and$\mu>0$ is the penalty parameter.

Since Eq. (2) is non-convex, the alternative optimization strategy is adopted, i.e., it updates one variable while fixing the others until the convergence criterion is reached. According to ADMM (Alternating Direction Method of Multipliers) algorithm, Eq. (2) is equivalent to four subproblems.

$\boldsymbol{P}^{\left[ \boldsymbol{e} \right]}\boldsymbol{,}\boldsymbol{P}^{\left[ \boldsymbol{s} \right]}$**-*Subproblem***: stACN first optimizes $P^{\left[ e \right]}$ and $P^{\left[ s \right]}$ by fixing the other variables and Eq. (2) is equivalent to the following subproblem:

${min}_{P^{\left[ e \right]},P^{\left[ s \right]}}\Phi\left( \mathcal{T}_{2}, P^{\left[ e \right]}X-P^{\left[ e \right]}XW^{\left[ e \right]}-E^{\left[ e \right]} \right)+\Phi(\mathcal{T}_{3},P^{\left[ s \right]}A-P^{\left[ s \right]}AW^{\left[ s \right]}-E^{\left[ s \right]})$ (3)

$s.t. P^{\left[ e \right]}\left( P^{\left[ e \right]} \right)^{'}=I,{P^{\left[ s \right]}\left( P^{\left[ s \right]} \right)}^{'}=I$.

According to Ref. [1], the optimal solution for $P^{\left[ e \right]}$ is deduced as $(P^{\left[ e \right]})'=U^{\left[ e \right]}(V^{\left[ e \right]})'$, where $U^{\left[ e \right]}$ and $V^{\left[ e \right]}$ are the left and right singular values of $(X-XW^{\left[ e \right]})(E^{\left[ e \right]}-\mathcal{T}_{2}/\mu)'$. Similarly, $P^{\left[ s \right]}$ is deduced as $P^{\left[ s \right]}=U^{\left[ s \right]}(V^{\left[ s \right]})'$, and $U^{\left[ s \right]}$ and $V^{\left[ s \right]}$ are the left and right singular values of $(A-AW^{\left[ s \right]})(E^{\left[ s \right]}-\mathcal{T}_{3}/\mu)'$ .

$\mathcal{W}$**-*Subproblem***: By fixing other variables, Eq. (2) with respect to $W^{\left[ e \right]}$ and $W^{\left[ s \right]}$ is reformulated as:

${min}_{W^{\left[ e \right]}}\Phi\left( \mathcal{T}_{2}, P^{\left[ e \right]}X-P^{\left[ e \right]}XW^{\left[ e \right]}-E^{\left[ e \right]} \right)+\Phi\left( \mathcal{T}_{1}^{\left[ e \right]},Z^{[e]}-W^{[e]} \right)$ (4)

${min}_{W^{\left[ s \right]}}\Phi\left( \mathcal{T}_{3}, P^{\left[ s \right]}A-P^{\left[ s \right]}AW^{\left[ s \right]}-E^{\left[ s \right]} \right)+\Phi\left( \mathcal{T}_{1}^{\left[ s \right]},Z^{[s]}-W^{[s]} \right)$ (5)

By setting the partial derivative of Eqs. (4) and (5) with respect to $W^{\left[ e \right]}$ and $W^{\left[ s \right]}$ to zero, we obtain the closed-form solution for $W^{\left[ e \right]}$ and $W^{\left[ s \right]}$as:

$W^{\left[ e \right]}=\left( \left( F^{\left[ e \right]} \right)^{'}F^{\left[ e \right]}+I \right)^{-1}\left( \frac{{(F^{\left[ e \right]})}^{'}T_{1}^{[e]}}{\mu}+\left( F^{\left[ e \right]} \right)^{'}F^{\left[ e \right]}-\left( F^{\left[ e \right]} \right)^{'}E^{\left[ e \right]}+Z^{\left[ e \right]}-\frac{W^{[e]}}{\mu} \right)$ (6)

$W^{\left[ s \right]}=\left( \left( F^{\left[ s \right]} \right)^{'}F^{\left[ s \right]}+I \right)^{-1}\left( \frac{{(F^{\left[ s \right]})}^{'}T_{1}^{[s]}}{\mu}+\left( F^{\left[ s \right]} \right)^{'}F^{\left[ s \right]}-\left( F^{\left[ s \right]} \right)^{'}E^{\left[ s \right]}+Z^{\left[ s \right]}-\frac{W^{[s]}}{\mu} \right)$ (7)

where $F^{\left[ e \right]}=P^{\left[ e \right]}X$,and$F^{\left[ s \right]}=P^{\left[ s \right]}A$.

$\boldsymbol{E}$**-*Subproblem***: By fixing other variables, Eq. (2) with respect to $E^{\left[ e \right]}$ and $E^{\left[ s \right]}$ is re-written as:

$${min}_{E}\lambda\left\| E \right\|_{2,1}+\Phi\left( \mathcal{T}_{2}, P^{\left[ e \right]}X-P^{\left[ e \right]}XW^{\left[ e \right]}-E^{\left[ e \right]} \right)$$

$+\Phi\left( \mathcal{T}_{3},P^{\left[ s \right]}A-P^{\left[ s \right]}AW^{\left[ s \right]}-E^{\left[ s \right]} \right)$ (8)

$$s.t. E=\left[ E^{\left[ e \right]};E^{\left[ s \right]} \right]$$

It can be further transformed into the equivalent problem as:

${min}_{E}\lambda\left\| E \right\|_{2,1}+\frac{1}{2}\left\| E-\mathcal{D} \right\|^{2}$ (9)

where $\mathcal{D}$ is constructed by vertically concatenating matrix $P^{\left[ e \right]}X-P^{\left[ e \right]}XW^{\left[ e \right]}+\mathcal{T}_{2}/\mu$ and $P^{\left[ s \right]}A-P^{\left[ s \right]}AW^{\left[ s \right]}+\mathcal{T}_{3}/\mu$.

According to Ref. [2], Eq. (8) is solved as:

$E_{:j}=\left\{ \begin{aligned} \frac{\left\| \mathcal{D}_{:j} \right\|_{2}-\frac{\lambda}{\mu}}{\left\| \mathcal{D}_{:j} \right\|_{2}}\mathcal{D}_{:j} if \left\| \mathcal{D}_{:j} \right\|_{2}>\frac{\lambda}{\mu} \\ 0 otherwise \end{aligned} \right.$ (10)

$\mathcal{Z}$**-*Subproblem***: By fixing $\mathcal{W}$, Eq.(2) with respect to $\mathcal{Z}$ is deduced as:

${min}_{\mathcal{Z}}\left\| \mathcal{Z} \right\|_{Ⓢ}$+$\Phi(\mathcal{T}_{1}\mathcal{,W-Z})$ (11)

It is equivalent to the optimization problem as:

${min}_{Z}\left\| \mathcal{Z} \right\|_{Ⓢ}+\frac{\mu}{2}\left\| \mathcal{Z+}\frac{\mathcal{T}_{1}}{\mu}\mathcal{-W} \right\|$ (12)

Ref. [3] proves that Eq. (12) is sovled with tensor tubal shrinkage operator. Specifically, the Lagrangian multipliers $\mathcal{T}_{1}$, $\mathcal{T}_{2}$, and $\mathcal{T}_{3}$ as well as the penalty parameter $\mu$ are updated as:

$\left\{ \begin{aligned} T_{2}=T_{2}+\mu(P^{\left[ e \right]}X-P^{\left[ e \right]}XW^{\left[ e \right]}-E^{\left[ e \right]}) \\ T_{3}=T_{3}+\mu(P^{\left[ s \right]}A-P^{\left[ s \right]}AW^{\left[ s \right]}-E^{\left[ s \right]}) \\ \mathcal{T}_{1}=\mathcal{T}_{1}+\mu(\mathcal{W-Z)} \\ \mu=min\{\rho\mu,\mu_{max}\} \end{aligned} \right.$ (13)

The complete procedure of our algorithm is summarized in Algorithm 1, and the convergence criterion (reconstruction error) is defined as $Re=\left\| P^{\left[ e \right]}X-P^{\left[ e \right]}XW^{\left[ e \right]}-E^{\left[ e \right]} \right\|_{F}^{2}+\left\| P^{\left[ s \right]}A-P^{\left[ s \right]}AW^{\left[ s \right]}-E^{\left[ s \right]} \right\|_{F}^{2}$.


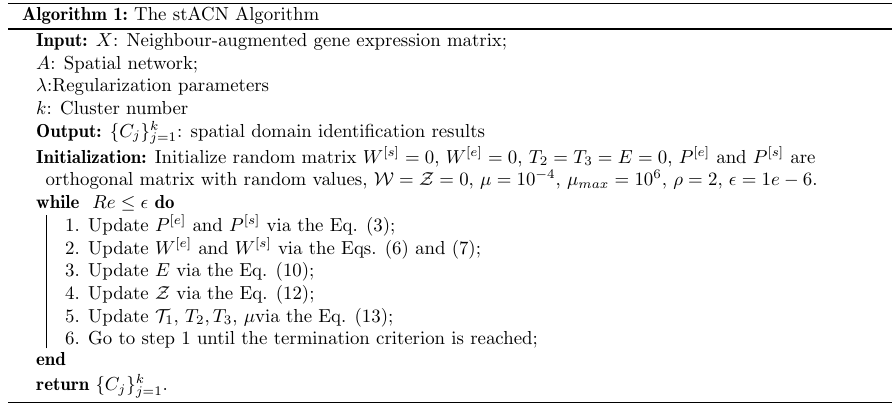


**(B) Algorithm analysis**

On the space complexity, the space for storing spatial transcriptomics data is $O(nm)$, where $n$ and $m$ are the number of cells and genes, respectively. The projection matrices need $O(nd)$, where $d$ represents the dimension. The coefficient matrices for self-representation learning need $O(n^{2})$. Because $m$ is 3,000 (top 3000 genes according to variance of expression), the overall space complexity of stACN is $O(n^{2})$, demonstrating that the proposed method is efficient in terms of space complexity.

On the time complexity, stACN mainly involves the iterative update of four variables. For each iteration, the complexity of updating $P^{\left[ e \right]}$ and $P^{\left[ s \right]}$ are $0(d^{2}n)$, where $d$ represents the feature dimension. The complexity of updating $W^{\left[ e \right]}$ and $W^{\left[ s \right]}$ are $O(n^{3})$ due to the matrix inversion, and updating $E$would costs about $O(n^{2})$. As for the $\mathcal{Z}$ subproblem, the main computation are calculating the 3D FFT and 3D inverse FFT of an $n\times2\times n$ tensor and $n$ SVDs of $n\times2\times$ metrices in the Fourier domain. considering $n\gg2$and $log(n)>2$ for SRT data, it takes $O(2n^{2}\log\left( n \right)+4n^{2})\approx O(n^{2}log(n))$ to update $\mathcal{Z}$. Thus, the final complexity of the proposed algorithm is $O(n^{3})$. Actually, cell expression and spatial networks $W^{\left[ e \right]}$ and $W^{[s]}$ are extra-ordinary sparse, whose time complexity approximates $O(n^{2})$. Thus, the running time of stACN approximates $O(n^{2}log(n))$.

**(C) Identification of spatial domains**

After obtaining the matrix $W^{\left[ e \right]}$ and $W^{\left[ s \right]}$, the affinity graph $S$ is constructed as $S=(\left\| W^{\left[ e \right]} \right\|+\left\| W^{\left[ e \right]} \right\|^{'}+ \left\| W^{\left[ s \right]} \right\|+\left\| W^{\left[ s \right]} \right\|^{'})/2$ since $S$ is unnecessarily symmetric. The Leiden algorithm is deployed on the affinity graph to obtain spatial domains.

**(D) Parameter selection**

Notice that there are four parameters involved in stACN, i.e., the number of features $d$, the number of clusters $k$, the graph denoising weight$\lambda$, and the Schatten-*p* norm parameter *p*, which controls the degree of low-rank approximation.. All these parameters are empirically selected if no prior information is available.

Fig A(**A**) depicts how ARI of stACN changes as the number of features increases from 10 to 300 on the DLPFC dataset, where the performance of stACN increases from 10 to 200, and then decreases if the number of features is larger than 250. There is a good reason to explain this tendency. When the number of features is small, the features of cells cannot fully represent spatial domains, resulting in an undesirable performance. If the number of features is too large, the redundancy of features decreases the performance of algorithms. stACN achieves the best performance when the number of features is 200. Notice that stACN is quite stable since performance changes subtly when the number of features$d\in[50,200]$. Fig A(**B**) illustrates how the regularization parameter$\lambda$affects the performance of stACN on the DLPFC dataset and non-Visium datasets, including osmFISH and STARmap, where ARI decreases as $\lambda$ increases from 0.001 to 100. Since the parameter $\lambda$ determines the importance of graph denoising, a large value of the parameter $\lambda$ enforces stACN to remove clean parts of the spatial transcriptomics data, resulting in a decrease in ARI. Thus, we set $\lambda$ =0.001, and kept it fixed across all datasets to ensure consistency and stability.

Fig F visualize spatial domains from the breast cancer data identified by stACN by varying the number of clusters from 9 to 20. Increasing the number of clusters from 13 to 20 results in over-segmentation of spatial domains. In other words, some small spatial regions (over-segmented from healthy regions) provide finer-granularity, but fail to provide additional meaningful distinctions. We set the number of clusters as 13.

To further assess the stability of the stACN under different Schatten-*p* norms, we conduct an ablation study using the 151675 slice of the DLPFC and the non-Visium osmFISH datasets as examples. Specifically, we vary *p* from 0.1 to 1.0 with an interval of 0.1 and report the corresponding ARI values. Note that when *p*=1.0, the tensor Schatten-*p* norm degenerates into the tensor nuclear norm. As illustrated in Fig A(**C**), the clustering performance varies noticeably with different values of *p*. In particular, the best performance is achieved when *p*=0.9 and *p*=0.8 on the DLPFC and osmFISH datasets, respectively. This demonstrates that *p* has a significant influence on the clustering results. This is because the parameter *p* affects how the model leverages the disparity among singular values. Moreover, the tensor Schatten-*p* norm provides a better approximation to the target rank when learning the consensus graph, thereby improving the robustness of the learned representations. Therefore, we set *p* = 0.9 in all experiments.

**(E) Detection of differentially expressed genes**

stACN performs differential expression analysis of genes (DEGs) for each spatial domain by using the Wilcoxon rank-sum test implemented in SCANPY package. Genes are expressed in 80% cells in each domain, and are with $\left| {log}_{2}(fold change) \right|\geq2$, and an FDR-adjusted p-value $\leq$ 0.05 are selected as DEGs. Gene enrichment analysis is performed with clusterProfiler [4].

**(F) Joint vs Separation of denoising and spatial domain identification**

stACN utilizes the conserved cells across various slices to correct spatial location of cells in each slice, forcing cells from various slices to be consistent, which effectively removes batch effect. In details, stACN first stacks multiple slices from various batches with SCANPY, and construct attributed cell network. Then, stACN learns the compatible cell features from the constructed network with tensor singular value decomposition, where consistent features of cell across different slices or batches are enhanced, thereby removing batch effect in feature space.

**(G) Acceleration of stACN with GPU**

On the 10$\times$ Genomics data, we independently execute each algorithm for each slide of DLPFC data, where distributions of running time and space of various algorithms are summarized in Fig R**(A1)** and (**A2)**, respectively. From Fig R(**A1)**, it is easy to conclude that Giotto and BayesSpace are slower than stLearn, stACN and MNMST, and SCANPY, SpaDo, BANKSY, and SpaGCN are faster than others. Fig R(**A2)** illustrates distributions of spaces for various algorithms for DLPFC data, where space complexity of Gitto and stLearn much higher than others. From Fig R(**A)**, it is easy to assert that

1. Gitto and stLearn are time- and space-consuming, hampering the applications of identification of spatial domains in SRT data. And, stLearn reduces running time by sacrificing space, whereas BayesSpace reduces space complexity by sacrificing running time.
2. SCANPY, SEDR and SpaGCN are efficient in terms of running time and space, whereas performance of these is undesirable. In other words, these algorithms improve efficiency by sacrificing accuracy of algorithms.
3. stACN reaches a good tradeoff between space and running time. Furthermore, it achieves the best performance on the identification of spatial domains, demonstrating that stACN provides an excellent alternative for current algorithms.

On the MERFISH data, we validate the efficiency of the algorithms by increasing the number of spots/cells from 1,000 to 20,000. Since MNMST is also a typical network-based model, and the BASS algorithm can perform multi-sample analysis, only MNMST, BASS, and stACN are selected for comparison. Fig R(**B1)** and (**B2)** describe the running time and space of algorithms with various sizes of data respectively, where MNMST reduces space complexity by sacrificing running time. These results demonstrate that stACN reaches a good balance between time and space with the best performance of identifying spatial domains.

Even though we demonstrate that stACN reaches a good balance between time and space with the best performance of identifying spatial domains, its efficiency is still undesirable since it fails to address large-scale ST data. To solve this problem, we accelerate it with graphic processing unit (GPU), i.e., accelerating stACN with hardware. The running time and space of stACN with CPU and GPU for DPLDC are summarized in Fig R(**C1)** and (**C2)** respectively, where stACN(GPU) accelerates 2$\sim$3 times by sacrificing extra 50% space, demonstrating that GPU is promising for improving applicability of stACN. The acceleration strategy is also efficient for MERFISH, where the improvement of time and space is shown in Fig R(**D1)** and (**D2)**, respectively.

**(H) Experimental Setup of Baselines**

1. **Sprod**: We adapted the Sprod workflow described in their online tutorial (https://github.com/yunguan-wang/SPROD). We conducted grid searches over the recommended parameter ranges, including R (0.04–0.24, step size = 0.01), K (3–10, step size = 1), U (250, 500 and 1,000), the Lambda (0.1–1, step size = 0.1, plus 5 and 20), and $L_{E}$ (0.3125, 0.625, 1.25 and 2.5).
2. **MIST**: We adapted the MIST workflow described in their online tutorial (<https://github.com/linhuawang/MIST.git>). We performed grid searches over the recommended parameter ranges, including threshold value (0.1-0.9, step size = 0.1), and sigma=0.1.
3. **DIST**: We adapted the DIST workflow described in their online code (<https://github.com/zhaoyp1997/DIST>). We ran the method with the recommended parameters, including K = 2 and L = 5.
4. **SCANPY**: Raw gene expression was preprocessed to select the top 3,000 HVGs and log-normalized. We then ran PCA to extract the top 30 principal components (PCs) and then the nearest neighbor network was constructed using the scanpy.pp.neighbor() function with default parameters. Finally, SCANPY obtains the clustering assignments using the scanpy.tl.louvain() function. For the DLPFC dataset, the resolution parameter was tuned manually to ensure the number of clusters matches the ground truth.
5. **Gitto**: We adapted the Giotto workflow described in their online tutorial (<http://spatialgiotto.rc.fas.harvard.edu/giotto.visium.brain.html>). Specifically, the expression data was normalized by applying the scalefactor parameter set to 6000. The spatial network was first constructed using the createSpatialNetwork() function with k=5 and maximum_distance_knn=1000. Then, the spatial domains were identified using the doHMRF() function with the parameter betas set to c(0, 10, 20).
6. **stLearn**: We adapted the stLearn workflow described in their online tutorial(https://stlearn.readthedocs.io/en/latest/stSME_clustering.html#Human-Brain-dorsolateral-prefrontal-cortex-(DLPFC). The stLearn.SME.SME_normalized() function was performed on the raw counts of all genes with parameters use_data=”raw” and weights=” physical_distance”. Then the first 30 PCs of the SME normalized matrix were used for further clustering and visualization.
7. **SEDR**: We ran SEDR for all experiments with its recommended parameters in the online tutorial (https://github.com/JinmiaoChenLab/SEDR/). Specifically, the parameter k was set to 10, and the number of epochs was set to 200.
8. **BayesSpace**: BayesSpace was applied to the DLPFC datasets as described in their online tutorial (https://github.com/edward130603/BayesSpace).The input is the top 15 PCs of the log-normalized expression of the top 2,000 HVGs. The nrep parameter was set to 50,000 and the gamma parameter was set to 3. We only ran it for 10×Visium data because its package did not support the calculation of Stereo-seq data directly.
9. **SpaGCN**: SpaGCN was applied to the DLPFC datasets as described in their online tutorial (<https://github.com/jianhuupenn/SpaGCN/blob/master/tutorial/tutorial.ipynb>). We ran these methods with the recommended parameters, including s=1, b=49, p=0.5, n_clusters=7 (depending on the number of spatial domains) and max_epochs=200, and set the number of clusters to match the ground truth.
10. **STAGATE**: We ran STAGATE for all experiments using the recommended parameters provided in the official tutorial (https://github.com/QIFEIDKN/STAGATE_pyG).
11. **BASS**: We ran BASS for all experiments using the recommended parameters provided in the official tutorial (https://github.com/zhengli09/BASS).
12. **SpaDo**: We ran SpaDo for all experiments using the recommended parameters provided in the official tutorial (https://github.com/bm2-lab/SpaDo).
13. **BANKSY**:We ran BANKSY for all experiments using the recommended parameters provided in the official tutorial ( https://github.com/prabhakarlab/Banksy_py).
14. **MNMST**: We ran MNMST for all experiments using the recommended parameters provided in the official tutorial (https://github.com/xkmaxidian/MNMST.ipynb). Specifically, We ran these methods with the recommended parameters, including $\lambda\in[1,80]$, and $\gamma\in[1,20]$ for 10 $\times$ Genomics data, $\lambda\in[10,30]$, and $\gamma\in[20,80]$ for MERFISH data, and $\lambda\in[40,80]$, and $\gamma\in[1,40]$ for STARmap data, respectively.
15. **STMGCN**: We ran STMGCN for all experiments using the recommended implementation available at https://github.com/sxj204/stmgcn. Specifically, we used a single graph convolution layer for both views and set the layer dimension to 32 across all datasets. The model is optimized using the Adam optimizer with learning rates $\mathrm{lr}\in\{0.001,0.0001\}$.
16. **Sensitivity and Robustness Analysis of stACN Pre-processing Settings**

To evaluate the robustness of stACN with respect to pre-processing settings, we conducted a systematic sensitivity analysis on both Visium (DLPFC) and non-Visium datasets (STARmap and osmFISH)(Fig S). Specifically, the analysis considers KNN neighborhood size (K=3,5,8,15), distance metrics (Euclidean vs. Cosine), and PCA/HVG choices (with vs. without PCA and HVG).

**KNN parameter sensitivity**: To evaluate the sensitivity of stACN to the KNN parameter, we varied the number of neighbors K from 3 to 15 (K = 3, 5, 8, 15) (Fig S**(A)**). The results indicate that the performance of stACN remains generally stable across this range, achieving the best clustering accuracy on the DLPFC dataset when K = 5 (ARI = 0.559$\pm$0.049), while slightly lower performance is observed for very small or large K values (e.g., ARI = 0.543$\pm$ 0.044 for K = 3 and ARI = 0.494$\pm$0.073 for K = 15), likely due to insufficient or excessive neighborhood smoothing. For non-Visium single-cell datasets, stACN attains near-optimal performance with STARmap at K = 5 (ARI = 0.602) and osmFISH at K = 8 (ARI = 0.658), demonstrating its robustness across different data modalities. Accordingly, we set the number of neighbors to K = 5 for 10×Genomics datasets and K = 8 for non-Visium single-cell datasets.

**Distance metric**: We further evaluate the impact of different distance metrics and find that both Euclidean and cosine distances yield comparable results (Fig S**(B)**). This indicates that the graph learning and denoising modules in stACN effectively mitigate the influence of distance metric choice, demonstrating the robustness of the method.

**Highly variable gene selection**: To assess the influence of highly variable gene (HVG) selection, we compare model performance between the HVG and non-HVG settings (HVG: 0.559$\pm$0.049, non-HVG: 0.553$\pm$0.052, Fig S(**C**)). The results indicate that stACN achieves consistently clustering accuracy under both conditions, demonstrating its robustness to gene selection strategies. Notably, for non-Visium datasets (STARmap and osmFISH), the number of genes is limited (e.g., 1020 for STARmap and 33 for osmFISH), and thus no gene selection is performed. For Visium datasets, we select the top 3000 HVGs based on prior studies[5,6].

**PCA pre-processing**: To assess the impact of PCA pre-processing, we compared the performance of stACN with and without PCA (Fig S(**D**)). For the DLPFC dataset, stACN achieved an ARI of 0.559$\pm$0.049 with PCA and 0.512$\pm$0.053 without PCA. Similarly, for the STARmap dataset, the ARI increased from 0.562 without PCA to 0.609 with PCA, indicating that PCA effectively reduces noise and improves clustering accuracy in spatial transcriptomics datasets. These findings demonstrate that PCA enhances the robustness of stACN, particularly for datasets characterized by high dimensionality or substantial measurement noise.

Overall, these analyses confirm that stACN is robust to variations in KNN neighborhood size, distance metric, HVG selection, and PCA pre-processing, reflecting its ability to reliably capture spatial and transcriptional patterns across diverse datasets.

Supplementary Table

**Table A. Statistics of all real SRT datasets used in this study**

| **Platform** | **Resolution** | **Distance** | **Tissue type** | **Section** | **Spots/Cells** | **K_num** | **Enhance_ratio** |
| --- | --- | --- | --- | --- | --- | --- | --- |
| **10**$\boldsymbol{\times}$ **Visium** | 55μm | 100μm | Human dorsolateral  prefrontal cortex (DLPFC) | 151507,  151508,  151509,  151510,  151669,  151670,  151671,  151672,  151673,  151674,  151675,  151676. | 4226,  4384,  4789,  4634,  3661,  3498,  4110,  4015,  3639,  3673,  3592,  3460. | 5 | 0.2 |
|  |  |  | Human breast cancer | V1 | 3798 |  |  |
|  |  |  | Mouse Brain | Coronal | 2702 |  |  |
|  |  |  |  | Posterior | 3353 |  |  |
| **STARmap** | Single-cell | **-** | Mouse visual cortex | **-** | 817 | 8 | 0.8 |
| **osmFISH** | Single-cell | **-** | Mouse cortex | **-** | 5328 | 8 | 0.8 |
| **Stereo-seq** | 0.22μm | subcellular | Mouse olfactory bulb | **-** | 19527 | 8 | 0.8 |
|  | 0.22μm | subcellular | Mouse embryos at E9.5 | **-** | 5913 | 8 | 0.8 |

**Table B. Parameter settings used in simulation experiments**

| **Purpose** | **Ground truth cluster** | **Cluster**  **merge probability** | **Sample**  **size** | **Latent code dimension** | **Feature dimension** | **Noise$\sigma$** |
| --- | --- | --- | --- | --- | --- | --- |
| Performance with Gaussian random noise of increasing variance | 10 | 0.7 | 1,000 | 30 | 500 | from 0 to 0.8 in increments of 0.2 |
| Performance as the number of ground-truth sub-populations increases | 6,10,15 | 0.7 | 1,000 | 30 | 500 | 0.1 |

**Table C. Clustering performance of spatial transcriptomics methods measured by ARI**

| Dataset | SpaGCN | BayesSpace | stLearn | STAGATE | stACN |
| --- | --- | --- | --- | --- | --- |
| 151507  151508  151509  151510  151669  151670  151671  151672  151673  151674  151675  151676 | 0.507  0.469  **0.503**  0.527  0.395  0.392  0.564  **0.598**  **0.593**  0.477  0.503  0.395 | 0.470  0.437  0.420  0.429  0.434  0.426  **0.732**  0.428  0.553  0.201  0.224  0.278 | 0.490  0.314  0.414  0.440  0.322  0.228  0.387  0.340  0.301  0.380  0.380  0.396 | 0.530  0.500  0.460  0.470  0.500  0.430  0.500  0.440  0.490  0.470  0.595  0.520 | **0.540**  **0.520**  0.500  **0.570**  **0.560**  **0.468**  0.581  0.530  0.590  **0.650**  **0.630**  **0.550** |
| Breast Cancer (13) | 0.600 | 0.487 | 0.670 | 0.489 | **0.702** |
| Breast Cancer (20) | 0.596 | 0.510 | 0.573 | 0.514 | **0.610** |
| STARmap | 0.462 | 0.279 | 0.281 | 0.510 | **0.599** |
| osmFISH | 0.443 | 0.355 | 0.161 | 0.480 | **0.658** |

**References**

1. C. Zhang, Q. Hu, H. Fu, P. Zhu, X. Cao, Latent multi-view subspace clustering. *In Proceedings of the IEEE conference on computer vision and pattern recognition*,4279-4287 (2017).
2. G. Liu, Z. Lin, S. Yan, J. Sun, Y. Yu, Y. Ma, Robust recovery of subspace structures by low-rank representation. *IEEE transactions on pattern analysis and machine intelligence*, 35(1), 171-184(2012).
3. W. Xia, Q. Gao, Q. Wang, X. Gao, C. Ding, D. Tao, Tensorized bipartite graph learning for multi-view clustering. *IEEE Transactions on Pattern Analysis and Machine Intelligence*, 45(4), 5187-5202(2022).
4. T. Wu, E. Hu, S. Xu, M. Chen, P. Guo, Z. Dai, G. Yu, clusterProfiler 4.0: A universal enrichment tool for interpreting omics data. The innovation, 2(3) (2021).
5. Dong K, Zhang S. Deciphering spatial domains from spatially resolved transcriptomics with an adaptive graph attention auto-encoder. *Nature communications*, 13(1): 1739 (2022).
6. Wang Y, Liu Z, Ma X. MuCST: restoring and integrating heterogeneous morphology images and spatial transcriptomics data with contrastive learning. *Genome Medicine*, 17(1): 21 (2025).

**Supplementary Figure**

**
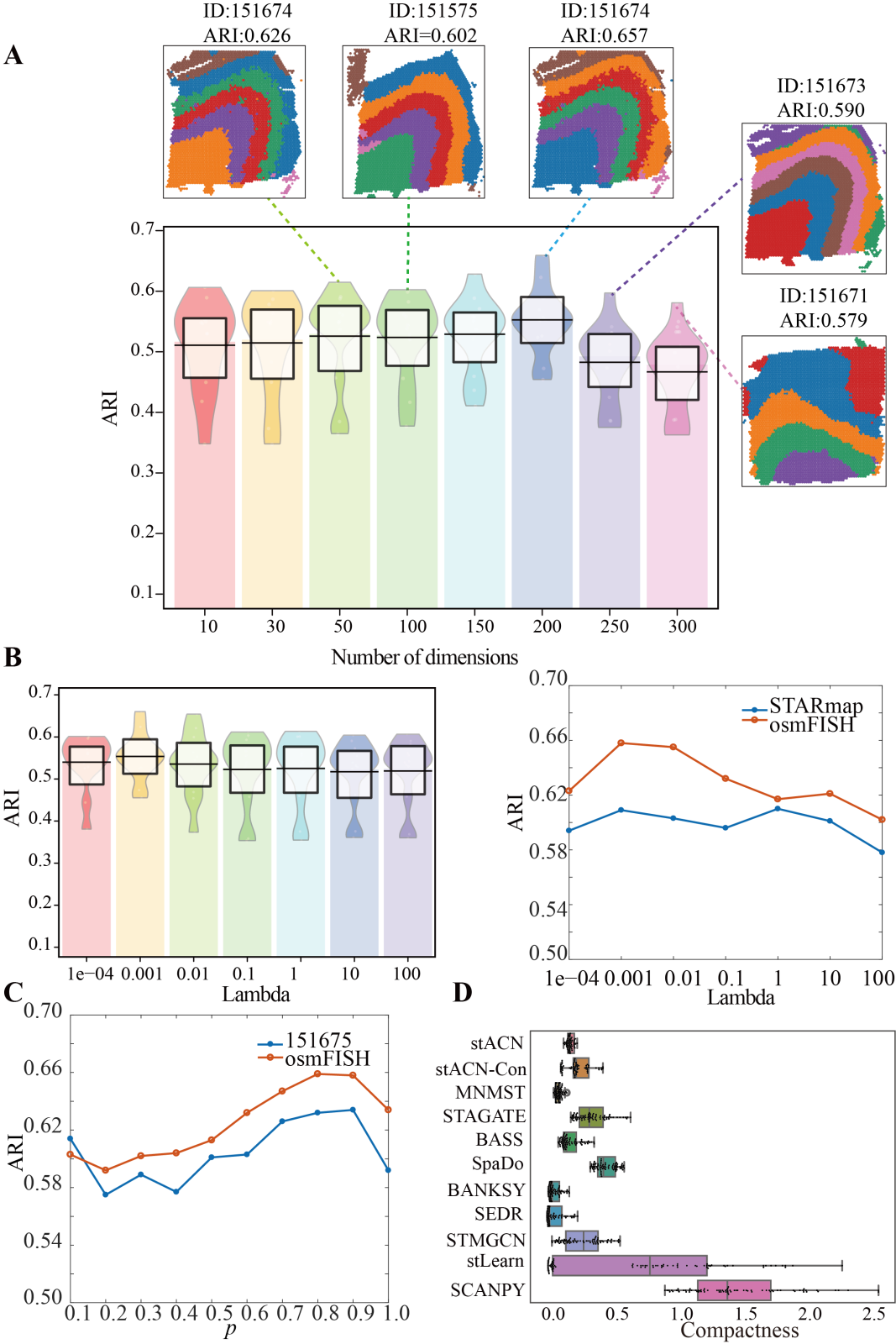
**

**Fig A.** Parameter selection of stACN. (**A**) Adjusted Rand Index (ARI) of stACN vs the number dimensions on the DLPFC dataset, (**B**) ARI of stACN vs the parameter$\boldsymbol{\lambda}$on the DLPFC dataset and non-Visium datasets, including osmFISH and STARmap, (**C**) ARI of stACN with respect to different tensor Schatten *p*-norms on the 151675 slice of DLPFC and the non-Visium osmFISH dataset, and (**D**) Distributions of compactness of spatial domains identified by various algorithms for DLPFC dataset identified by various aglorithms, which is sum of distances for each spot to the center of the corresponding clusters.


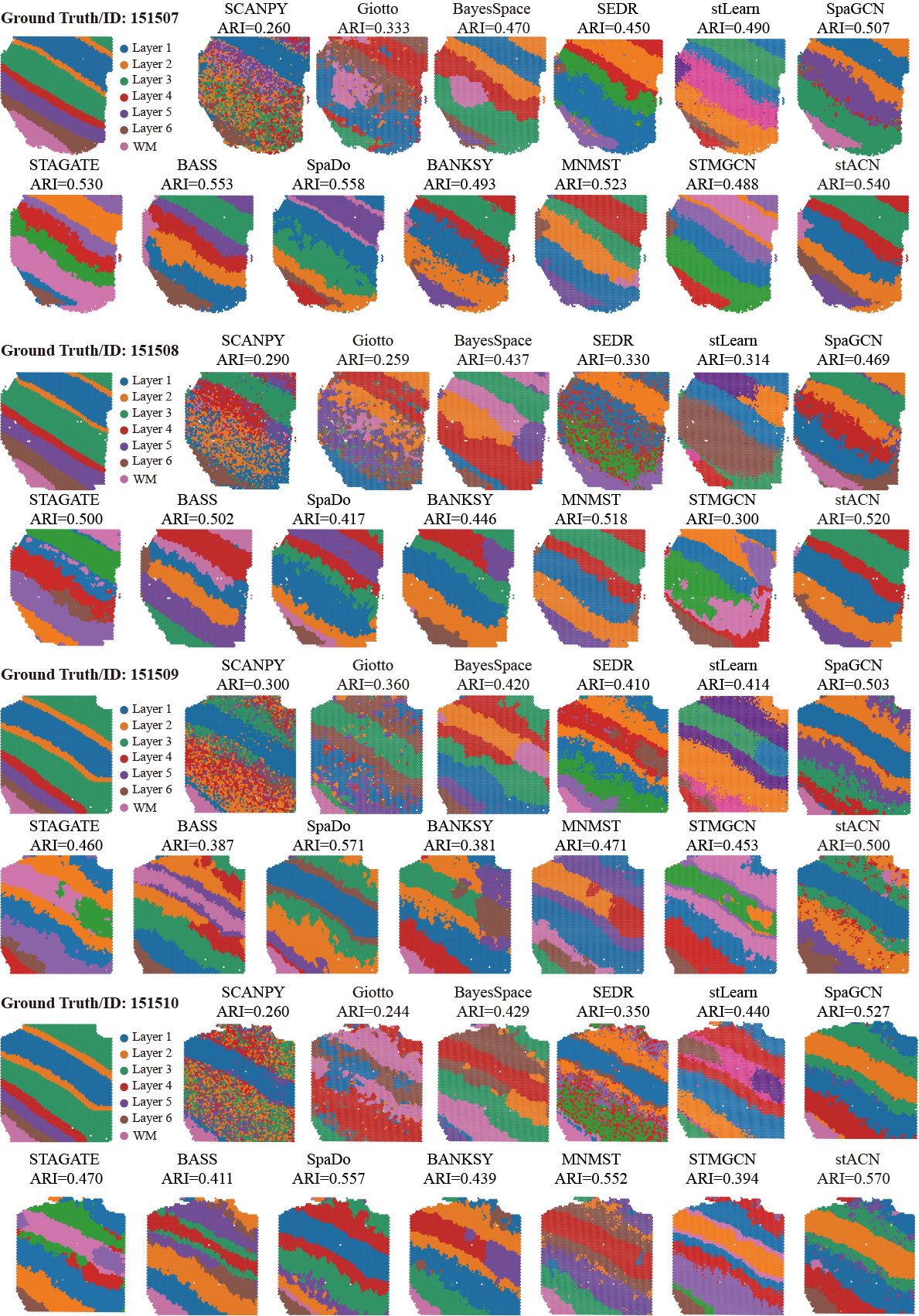


**Fig B.** Performance of various algorithms for spatial domain identification on annotated dorsolateral prefrontal cortex (DLPFC, http://spatial.libd.org/spatialLIBD) data (151507, 151508, 151509, 151510), where ground truth spots are mapped on their spatial location, divided into various cortical layers (L1-L6) and white matter (WM) layer.


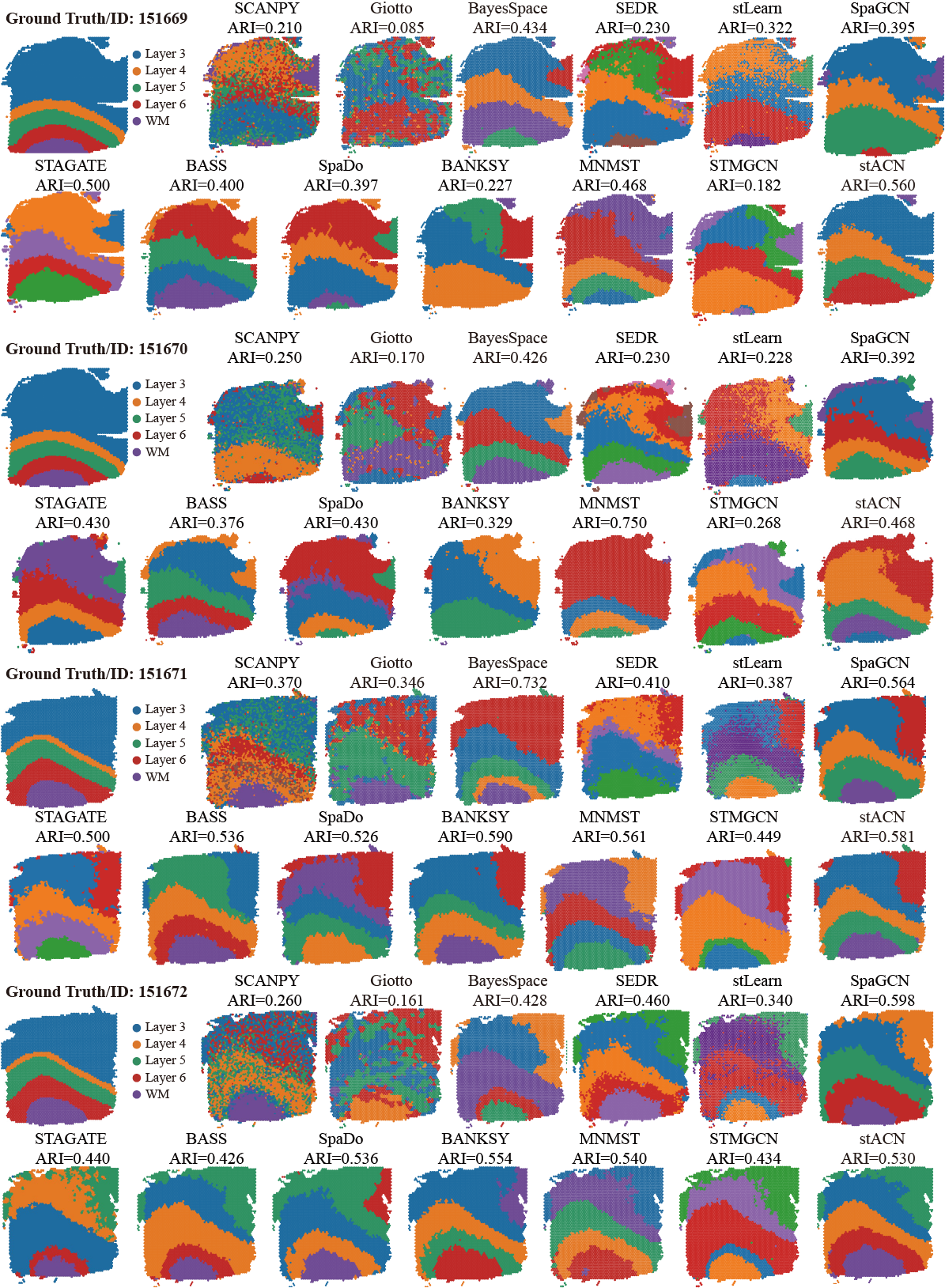


Fig C. Performance of various algorithms for spatial domain identification on Annotated dorsolateral prefrontal cortex (DLPFC, http://spatial.libd.org/spatialLIBD) data (151669, 151670, 151671, 151672), where ground truth spots are mapped on their spatial location, divided into various cortical layers (L3-L6) and white matter (WM) layer.


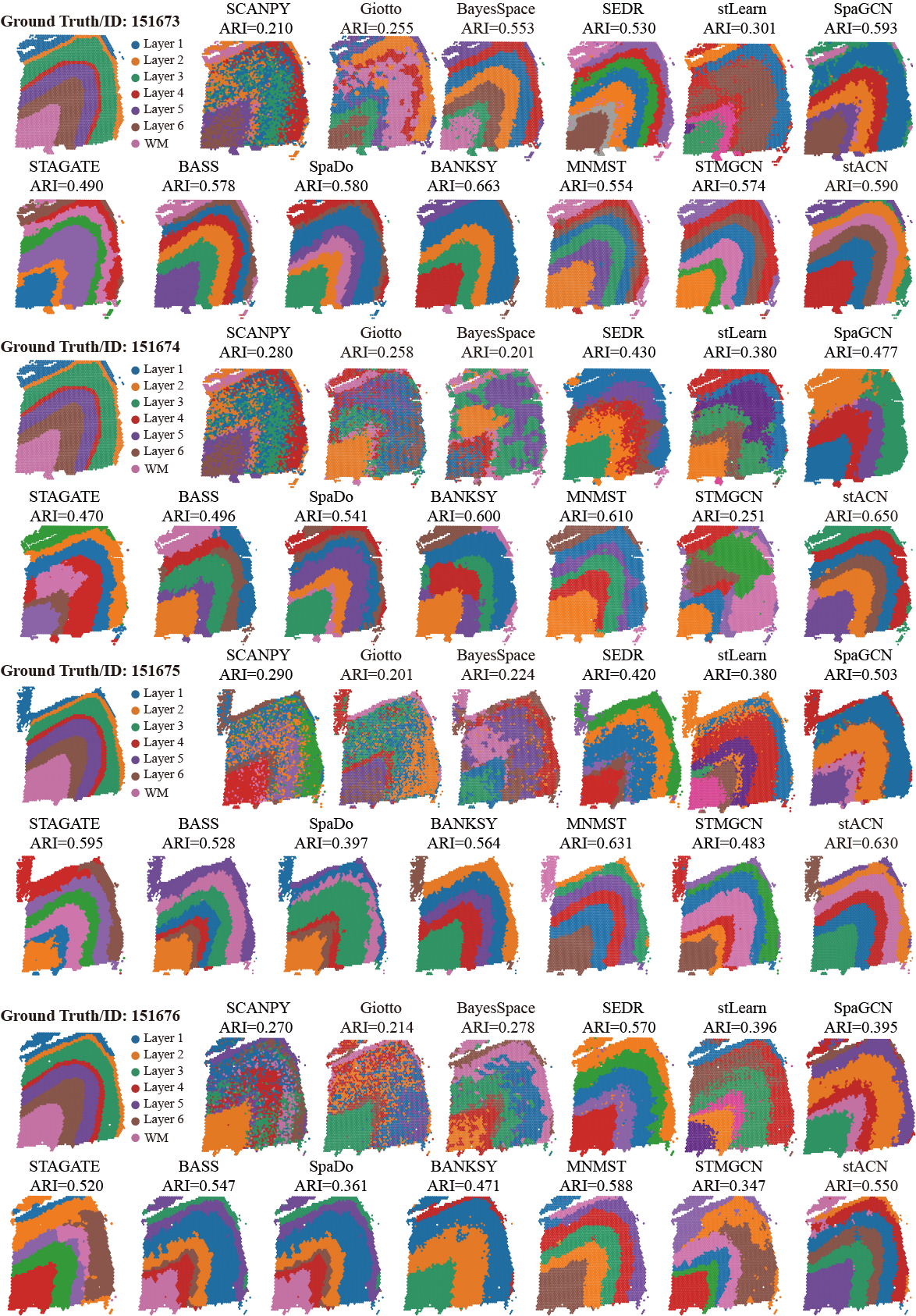


Fig D. Performance of various algorithms for spatial domain identification on Annotated dorsolateral prefrontal cortex (DLPFC, http://spatial.libd.org/spatialLIBD) data (151673, 151674, 151675, 151676), where ground truth spots are mapped on their spatial location, divided into various cortical layers (L1-L6) and white matter (WM) layer.


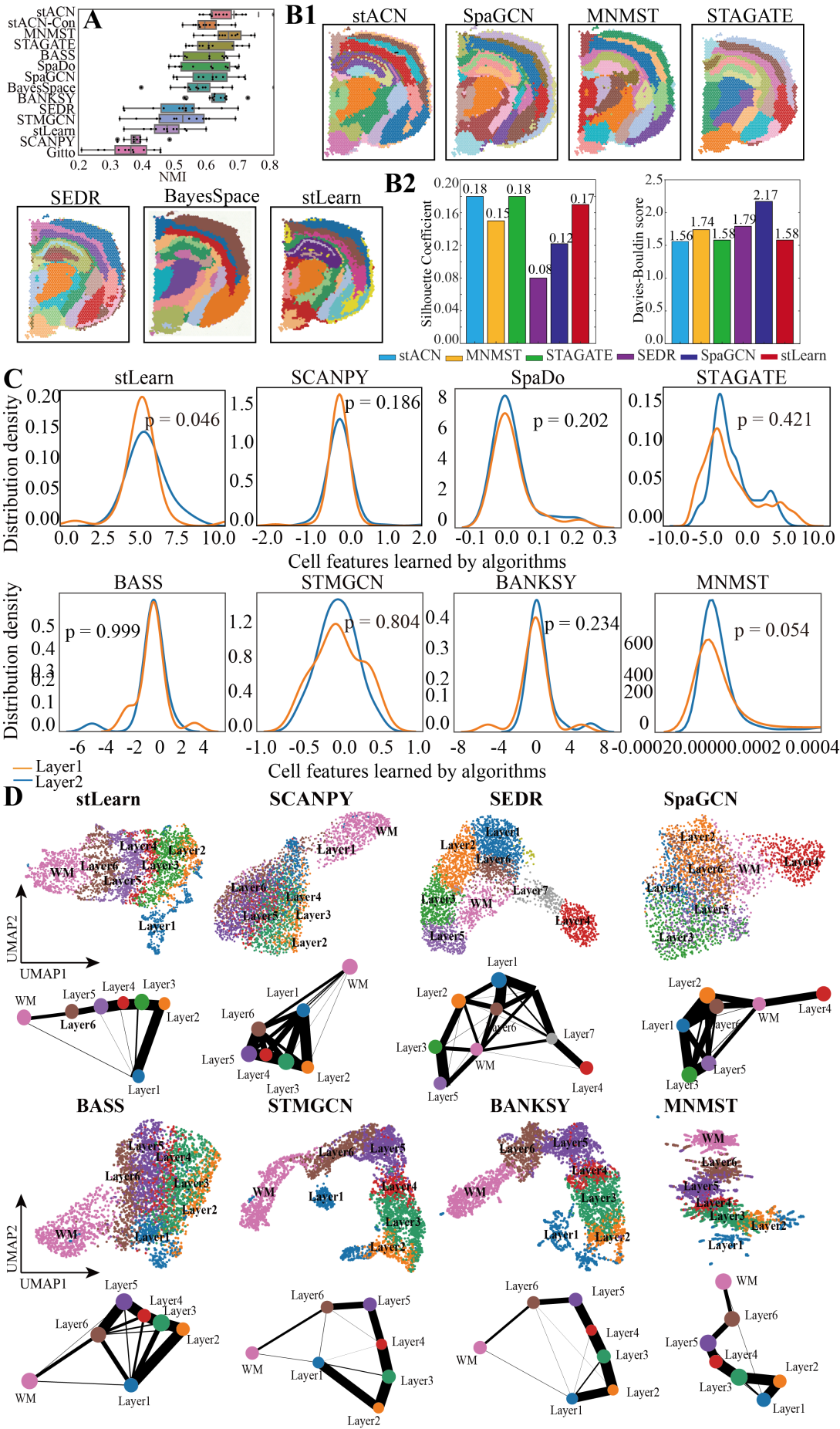


**Fig E. (A)** NMIs of various algorithms for identifying spatial domains in DLPFC dataset, where x-axis represents ARI (the center line, box limits and whiskers denote the median, upper and lower quartiles, and 1.5×interquartile range, respectively), **(B)** Spatial domains identified by stACN, MNMST, STAGATE, SpaGCN, BayesSpace, stLearn, and SEDR in mouse brain coronal, respectively **(B1)**. Histograms of Silhouette Coefficient (SC) and Davies-Bouldin (DB) scores for spatial domains identified by various algorithms for mouse brain coronal data **(B2)**. **(C**) Distribution density estimation between Layer1 and Layer2 in slice 151675 of DLPFC data with features of cells learned by various algorithms, and (**D**) UMAP visualizations of PAGA graphs of slice 151675 for various algorithms.


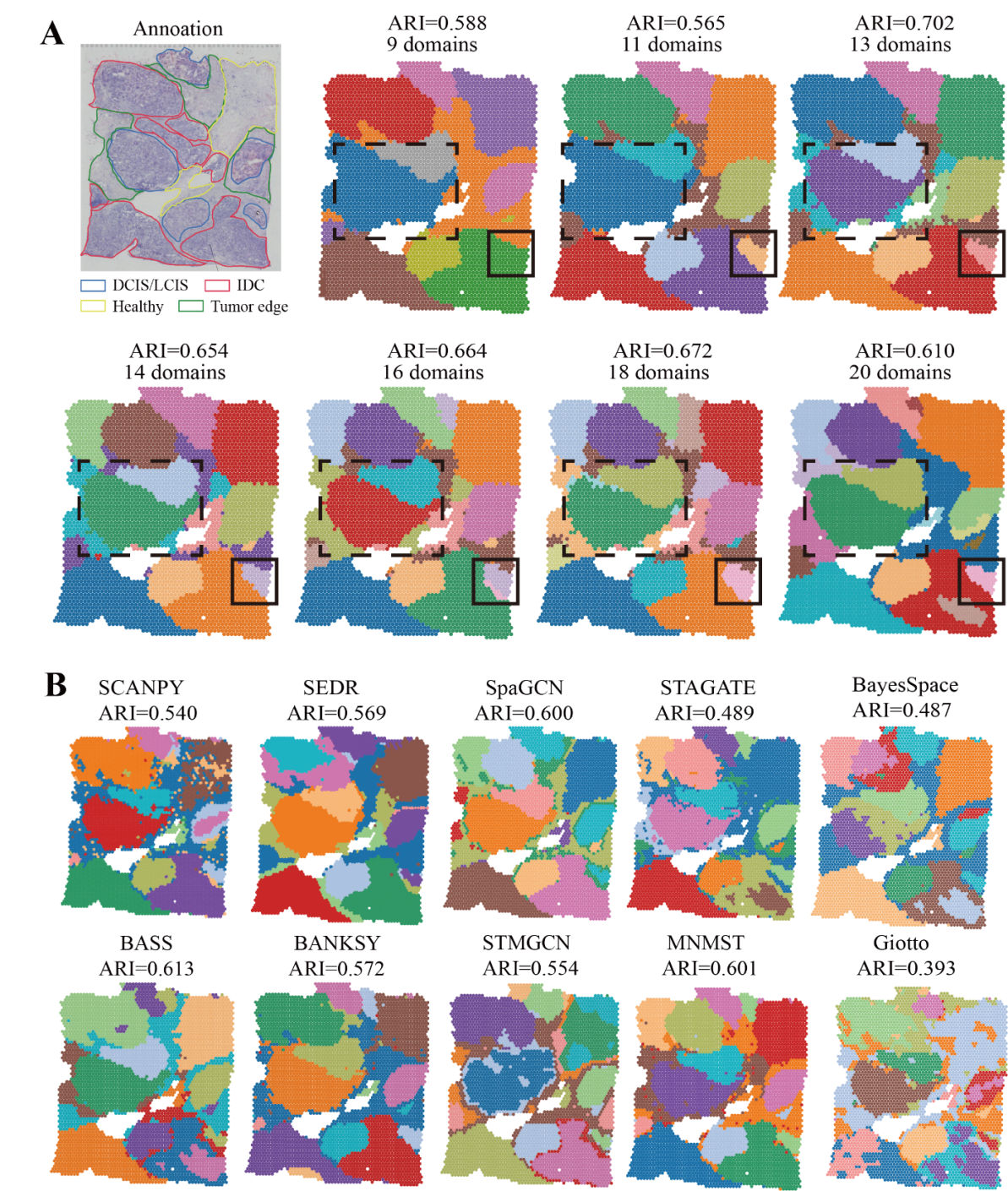


**Fig F.** Parameter selection of stACN on the breast cancer dataset. (**A**) Visualization of spatial domains identified by stACN with various number of clusters, where regions surrounded by black squares are spatial domains that are likely to be over-segmented. (**B**) Visualization of cancer spatial domains identified by various algorithms.


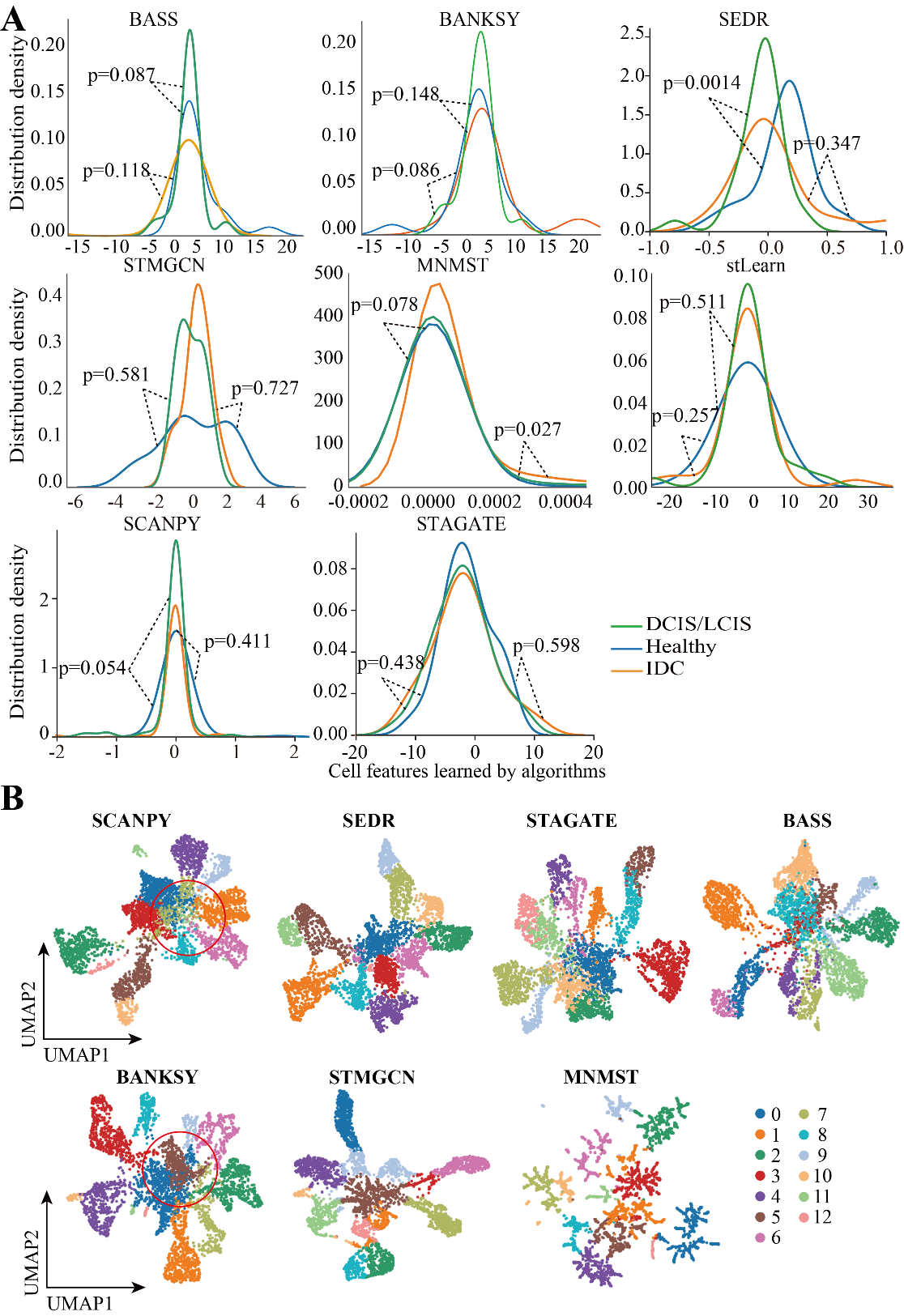


**Fig G.** stACN accurately identifies of cancer and non-cancer spatial domains. (**A**) Distribution density estimation of cells in IDC, DCIS/LCIS and Healthy domain in terms of the learned cell features, where x-axis denotes cell features, and Kolmogorov-Smirnov test is for significance. (**B**) UMAP visualization of spatial domains identified by stACN (left) and stLearn (right), where dashed circle denotes mixed domains.


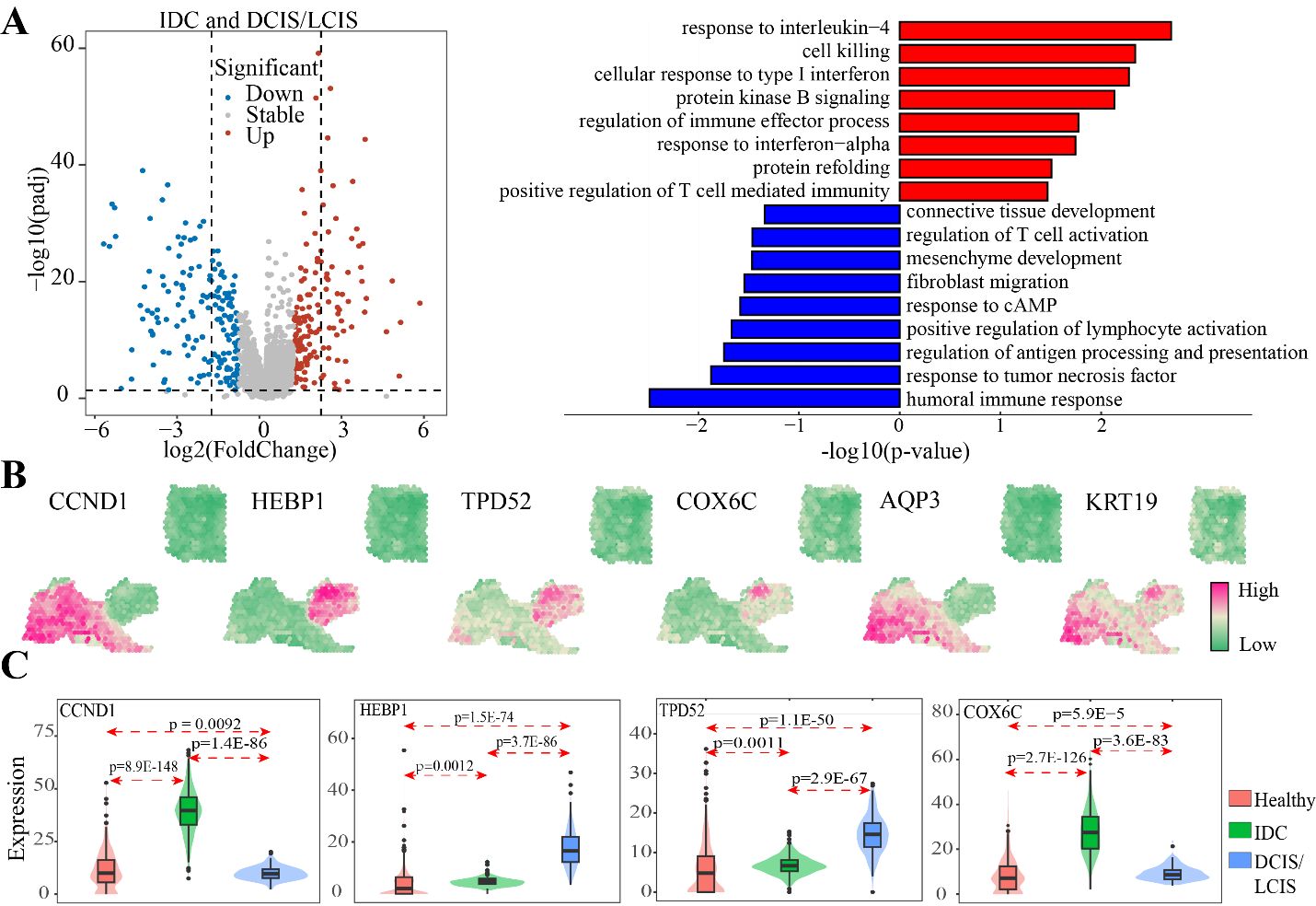


**Fig H.** Differential expression analysis between IDC and DCIS/LCIS domain in human breast data. (**A**) Volcano graph of differentially expressed genes (DEGs) between domains IDC and DCIS/LCIS, where x-axis denotes log2(Fold Change) and y-axis represents -log10(padj), where p-value is calculated with Student’s t-test (left). Gene ontology enrichment analysis of DEGs between domains IDC and DCIS/LCIS, where red denotes functions enriched by up-regulated genes, and blue by down-regulated ones (right). (**B**) Spatial distribution of expression of DEGs between IDC and DCIS/LCIS. (**C**) Distributions of expression levels of DEGs (|log fold change|$\geq$2) between IDC and DCIS/LCIS with wilcoxon rank-sum test.


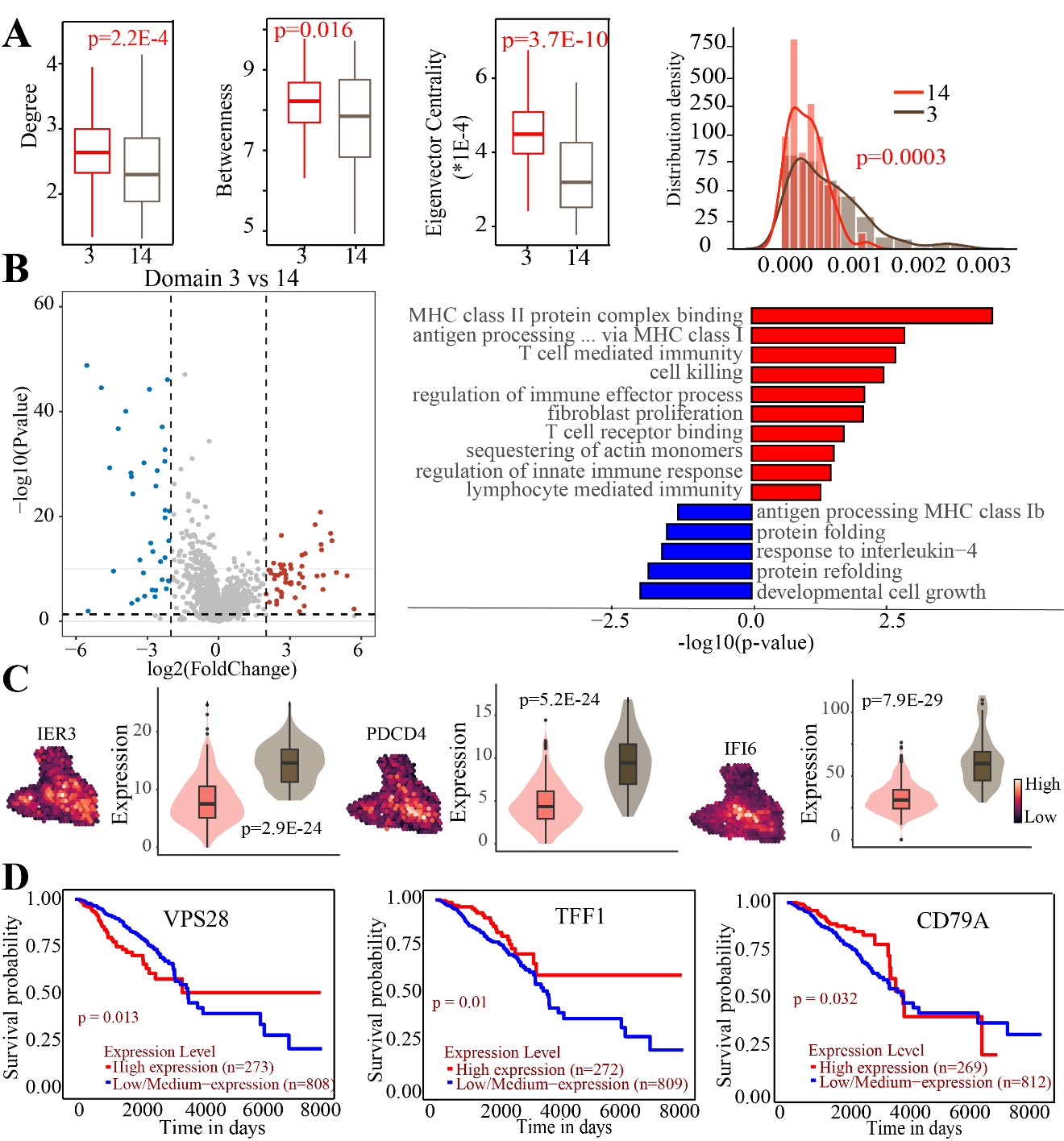


**Fig I.** Performance of various algorithms for spatial domain identification for human breast spatial transcriptomics data. (**A**) Distributions of degree, betweenness and eigenvector centrality of cells in domain 3 and 14 identified by stACN, where p-value is calculated with Student’s t-test (left). Distribution density estimation of features for cells in domain 3 and 14, where x-axis denotes cell features, and Kolmogorov-Smirnov test is for significance (right). (**B**) Volcano plot of DEGs, where x-axis denotes log2(Fold Change), and y-axis represents -log10(padj-value) (left). Biological functions significantly enriched by up- (red) and down-regulated (blue) DEGs, where x-axis denotes -log10(padj-value) (hypergeometric test for significance, right). (**C**) Spatial distribution of expression of IER3, PDCD4, and IFI6 with regional annotation (left), and Violin plots of gene expression (right). (**D**) DEGs associated with survival time of patients using Kaplan-Meier survival analysis (log-rank for significance).


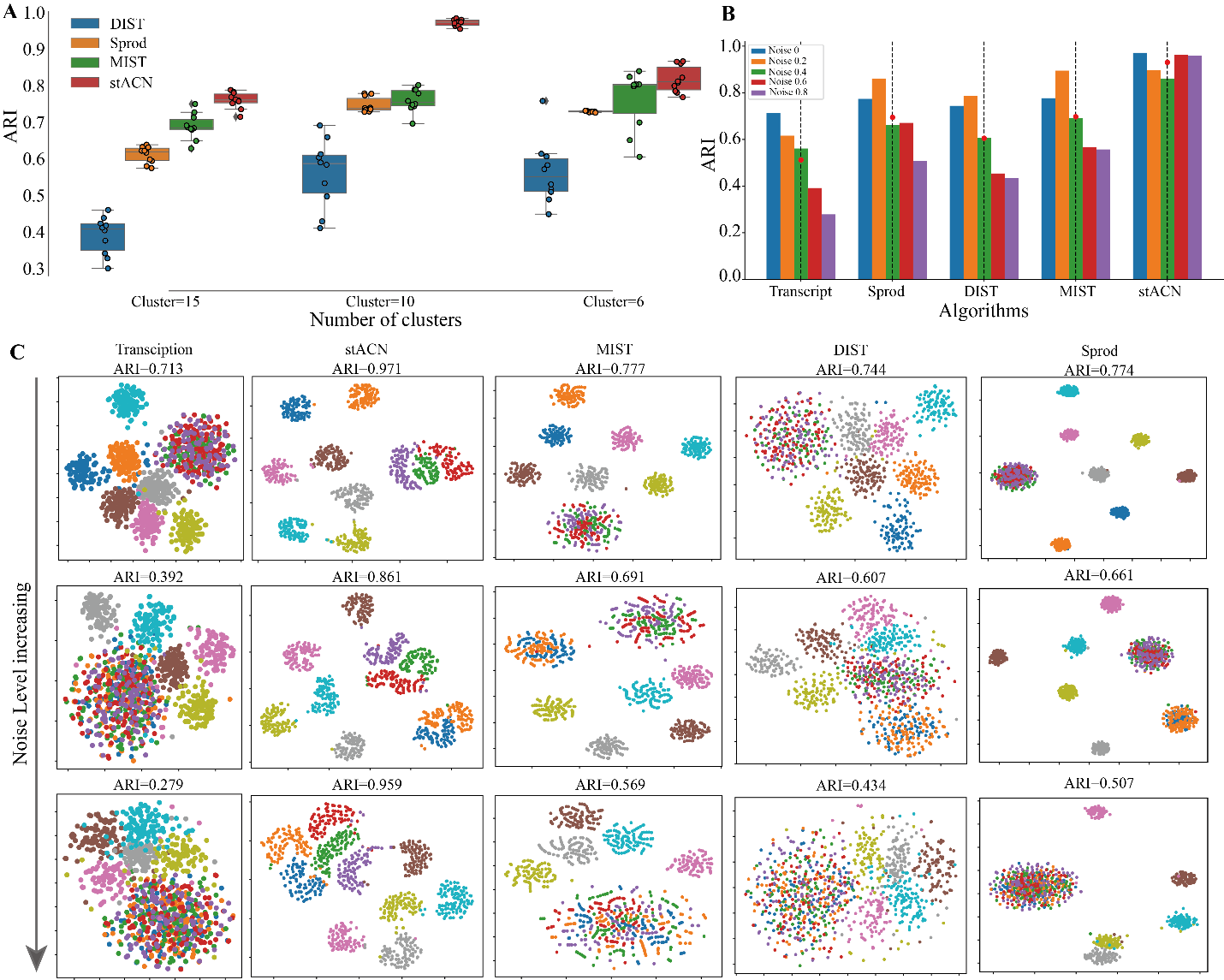


**Fig J.** Performance of various algorithms for denoising on the simulated data. (**A**) Distributions of ARIs of various algorithms on denoising simulated data with various number of clusters. (**B**) Boxplots of ARIs of various algorithms with various level of noise in simulated data, where the red point denotes the mean of ARIs for each algorithm. (**C**) Visualization of clusters obtained by various algorithms on the simulated data with various levels of noise.


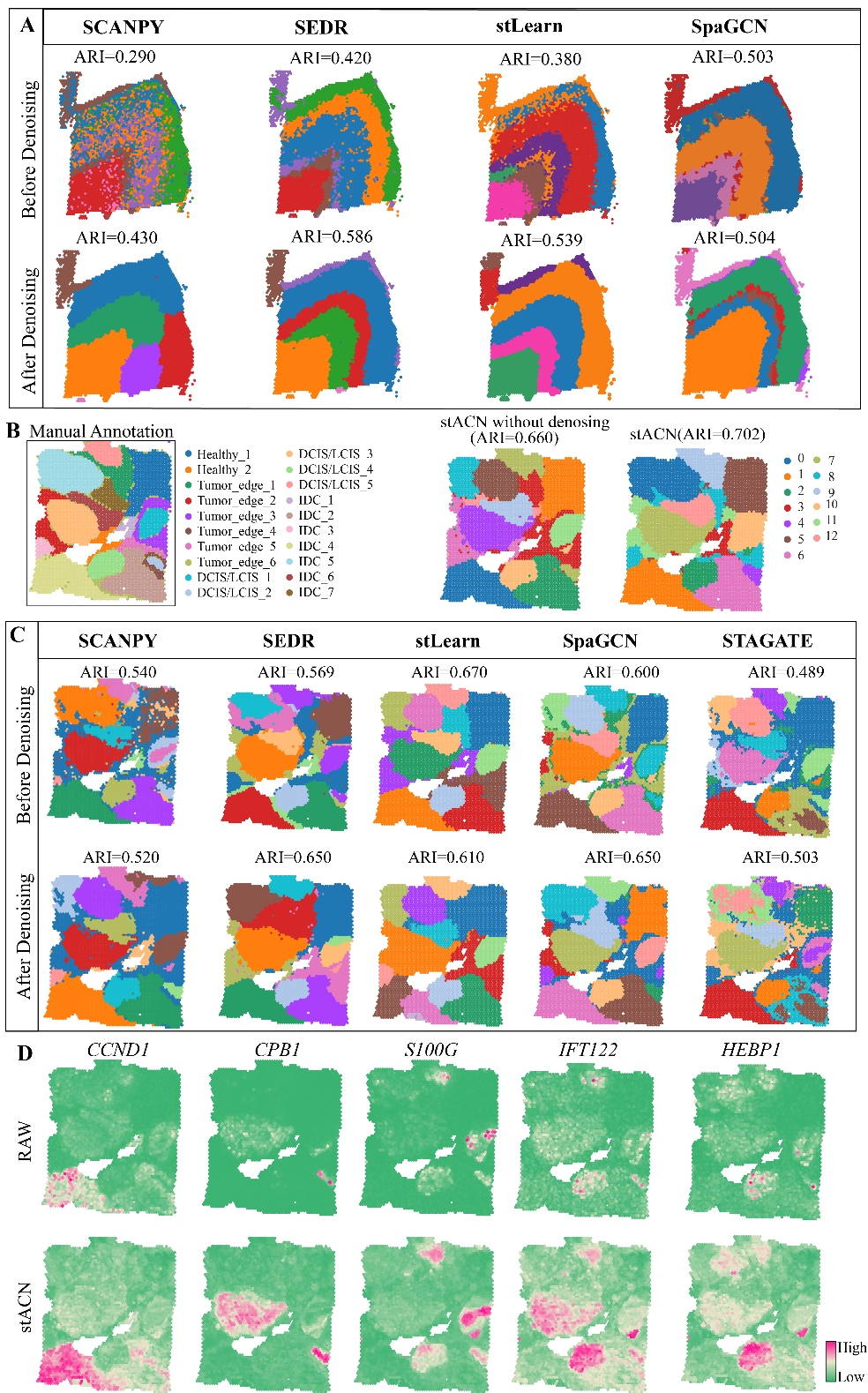


**Fig K.** stACN provides an effective and efficient graph denoising strategy for ST data. (**A**) Spatial domains identified by SCANPY, SEDR, stLearn, and SpaGCN for slices 151675 in DLPFC data before and after graph denoising. (**B**) Manual annotation for human breast cancer data (left), and visualization of spatial domains identified by stACN without and with denoising (right). (**C**) Spatial domains identified by various algorithm with and without graph denoising of stACN. (**D**) Visualizations of the original (up) and denoised ST data (bottom) for layer-marker genes.


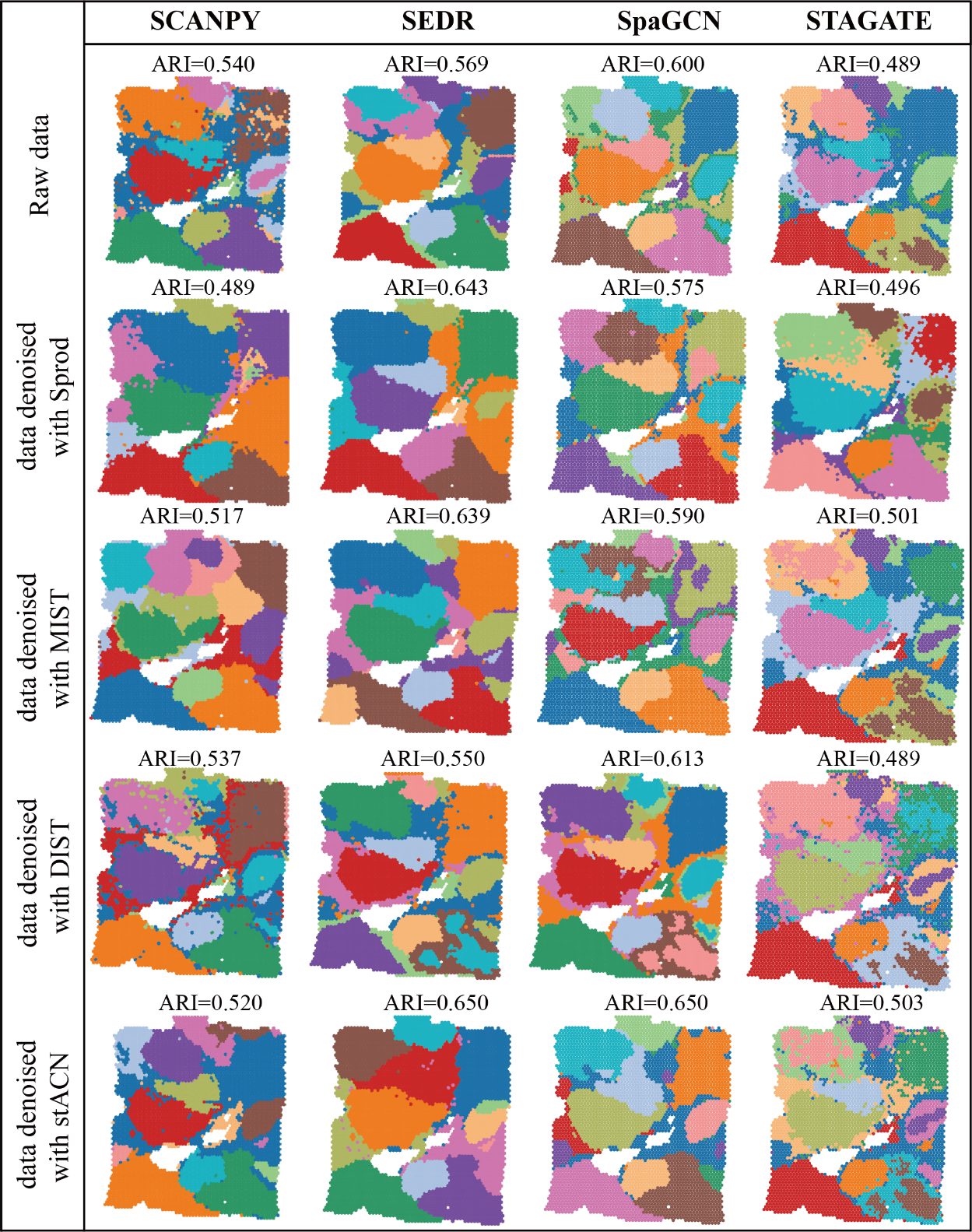


**Fig L.** Performance of algorithms with the separation strategy on the breast cancer dataset. Visualization of spatial domains identified by various algorithms with variants of datasets, where each column denotes an algorithm, and each row corresponds visualization of spatial domains identified by various algorithms with the raw and denoised data with various approaches, such as Sprod, MIST, DIST, and stACN, respectively.


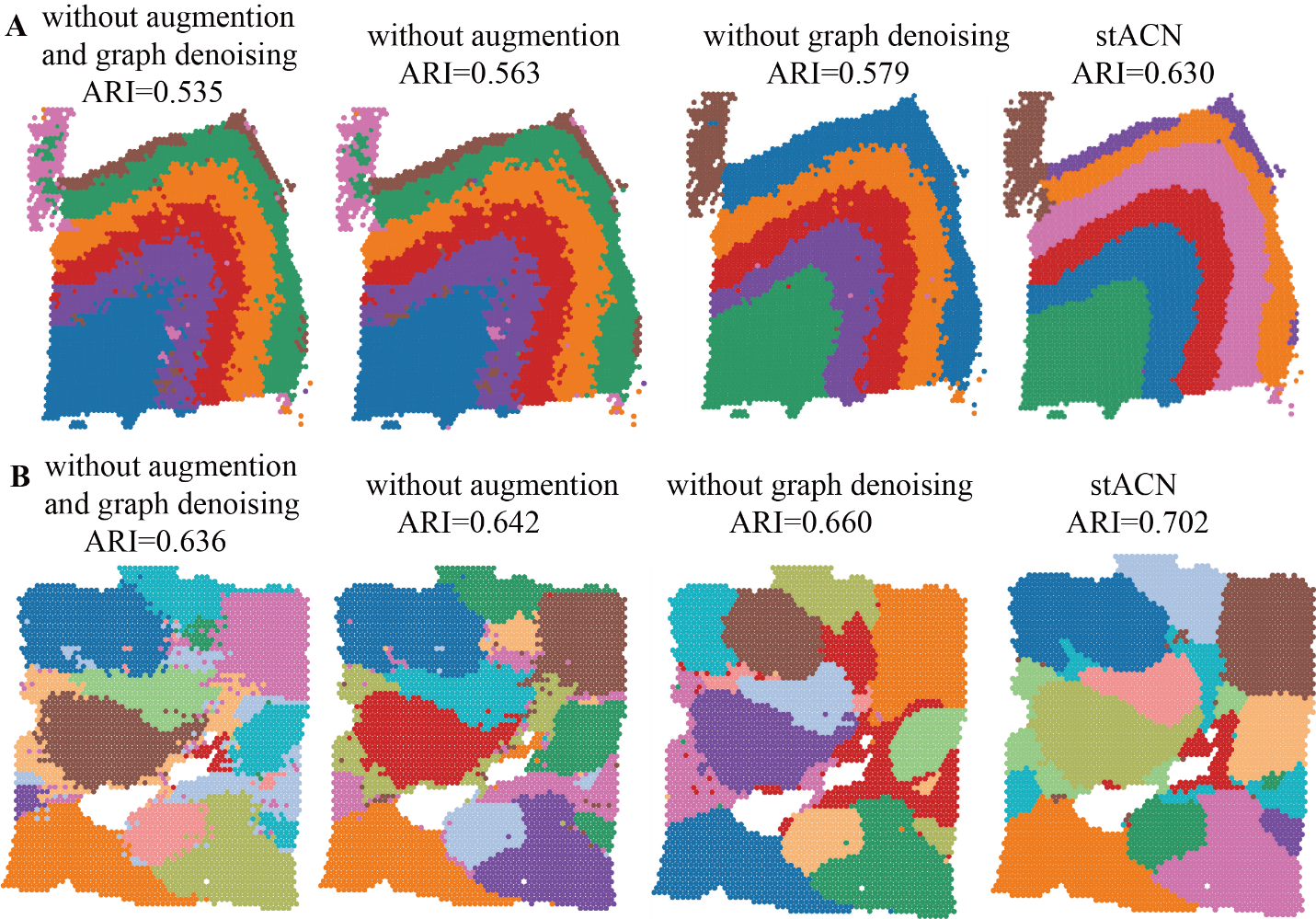


**Fig M.** Ablation analysis of stACN with various datasets. (**A**) Visualization of spatial domains identified by variants of stACN, i.e., without augmentation and graph denoising, without augmentation, without graph denoising, and stACN, on the slice 151675 of DLPFC dataset. (**B**) Visualization of spatial domains identified by variants of stACN for breast cancer dataset.


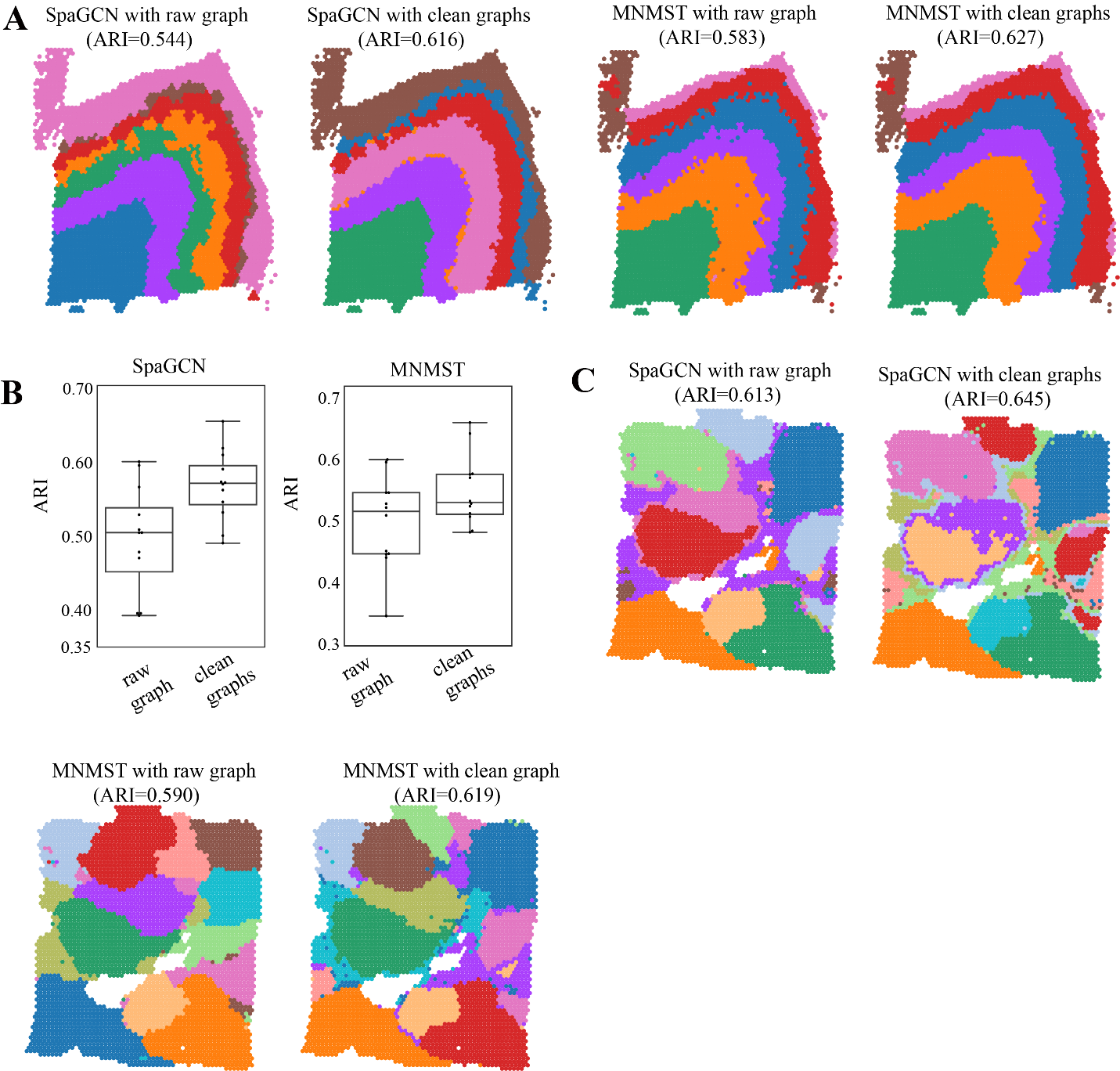


**Fig N.** Clean graphs obtained by stACN also enhance performance of network-based algorithms for spatial domain identification in spatial transcriptomics data. (**A**) Visualization of spatial domains identified by MNMST and SpaGCN with the raw (obtained by the raw spatial transcriptomics data) and clean (obtained by stACN) graphs on the slice 151675 of DLPFC data. (**B**) Distributions of ARIs of various algorithms with the raw and clean graphs for identifying spatial domains in DLPFC data. (**C**) Visualization of spatial domains identified by MNMST and SpaGCN with the raw and clean graphs on the breast cancer data.


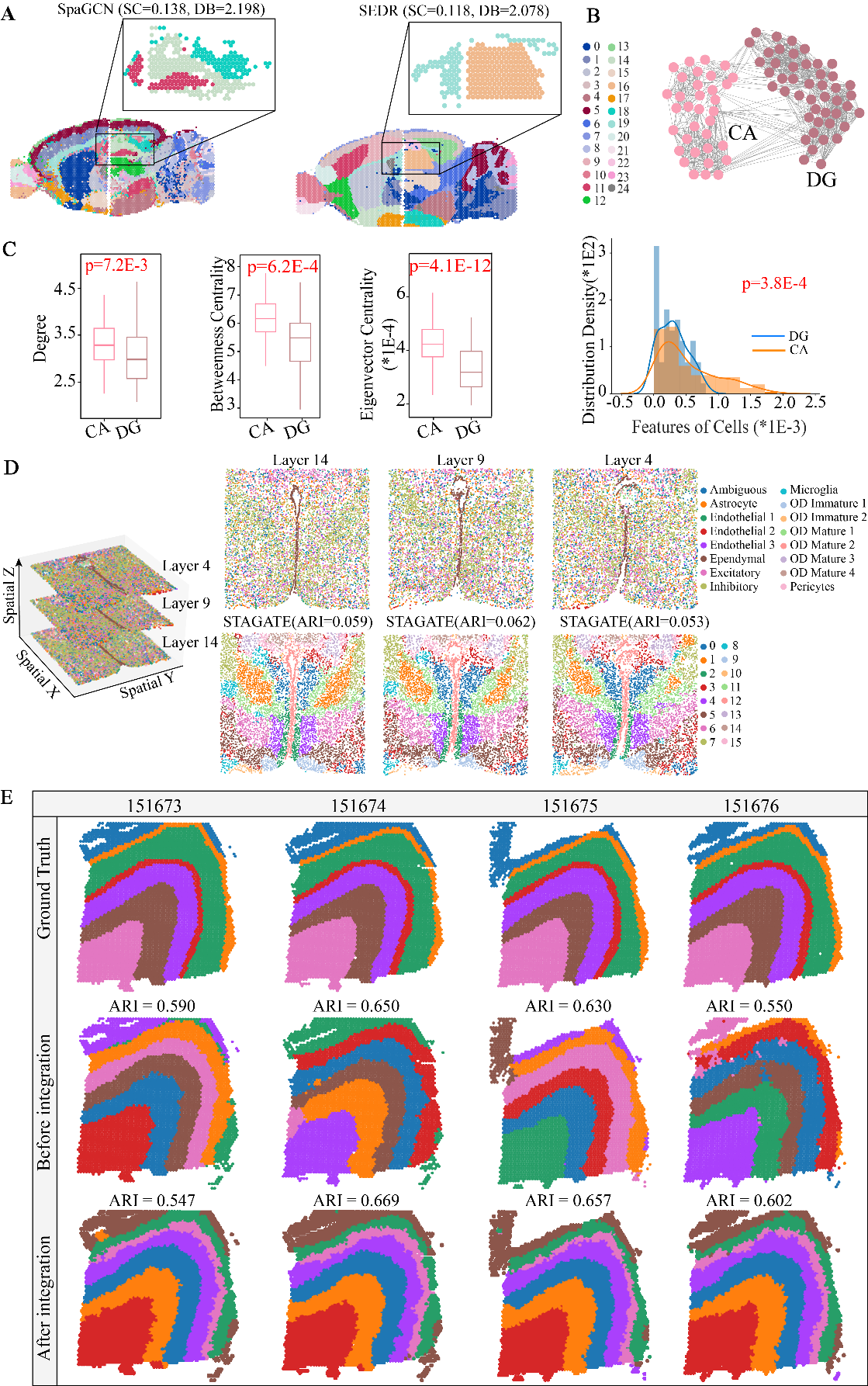


**Fig O.** Performance of stACN on integration analysis of spatial transcriptomics data. (**A**) Spatial domains identified by SpaGCN (left) and SEDR (right) in horizontally aligned mouse brain samples. (**B**) Topological structure of cells in cornu ammonis (CA) and dentate gyrus (DG) domains. (**C**) Distribution of degrees centrality of cells, betweenness, and eigenvector centrality in CA and DG domains (Student’s t-test for significance, left). Distribution density estimation of cells in CA and DG domains in terms of the learned cell features, where x-axis denotes cell features, and Kolmogorov-Smirnov test is for significance (right). (**D**) Performance of stACN on vertical integration of spatial transcriptomics data, manual annotation (up), and performance of STAGATE (bottom) on the 3D coordinates of MERFISH data. (**E**) Spatial domains identified by stACN for slices 151673, 151674, 151675, and 151676 in DLPFC data before and after integration.


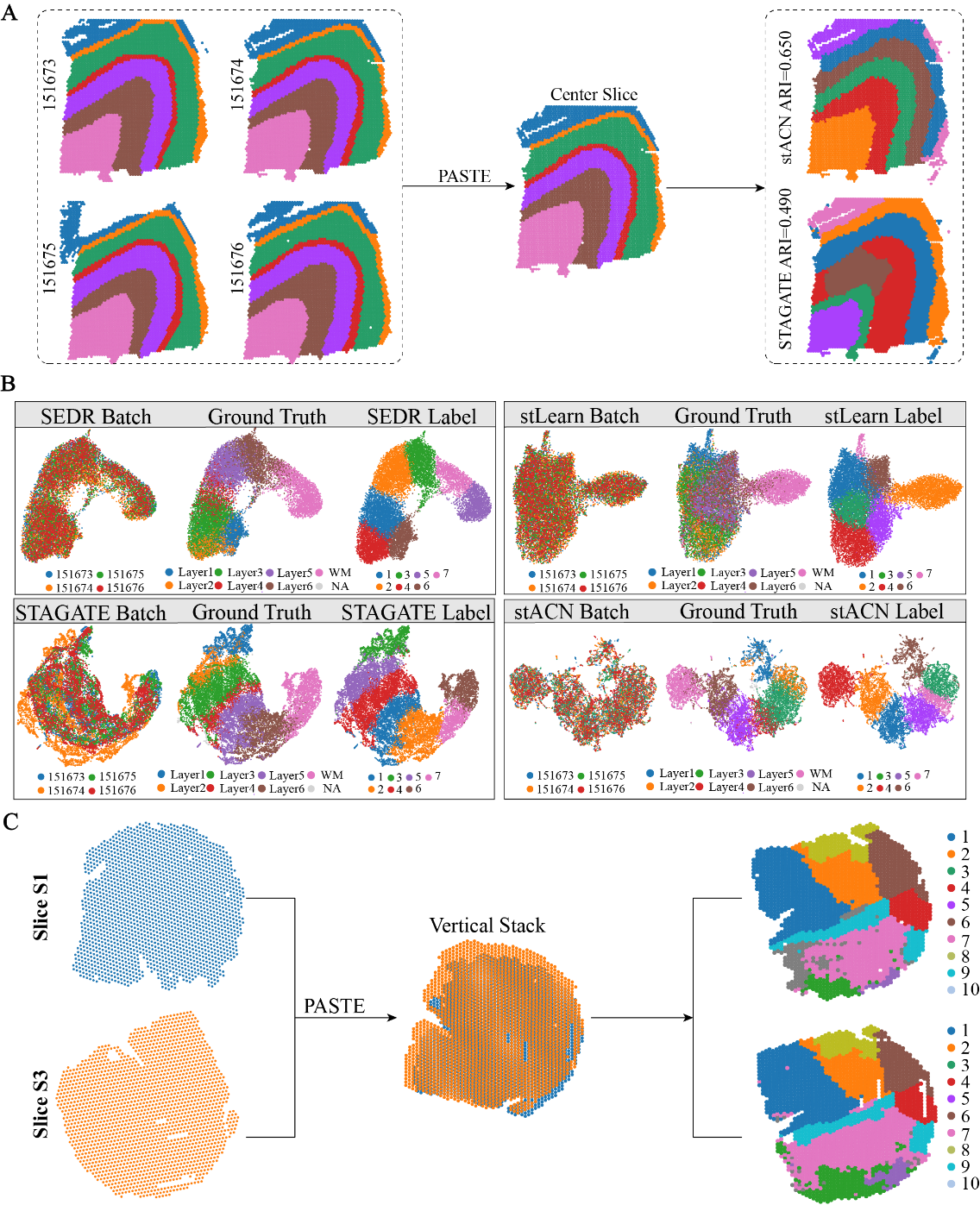


**Fig P.** Performance of stACN on integration analysis of spatial transcriptomics data. (**A**) Performance of stACN and STAGATE on the center slice generated by PASTE. (**B**) UMAP plots of integrated spatial transcriptomics with various algorithms, including stACN, STAGATE, stLearn and SEDR, for slices 151673, 151674, 151675, and 151676 in DLPFC dataset. (**C**) Performance of stACN for removing batch effect and noise on the vertical stack data generated by PASTE.


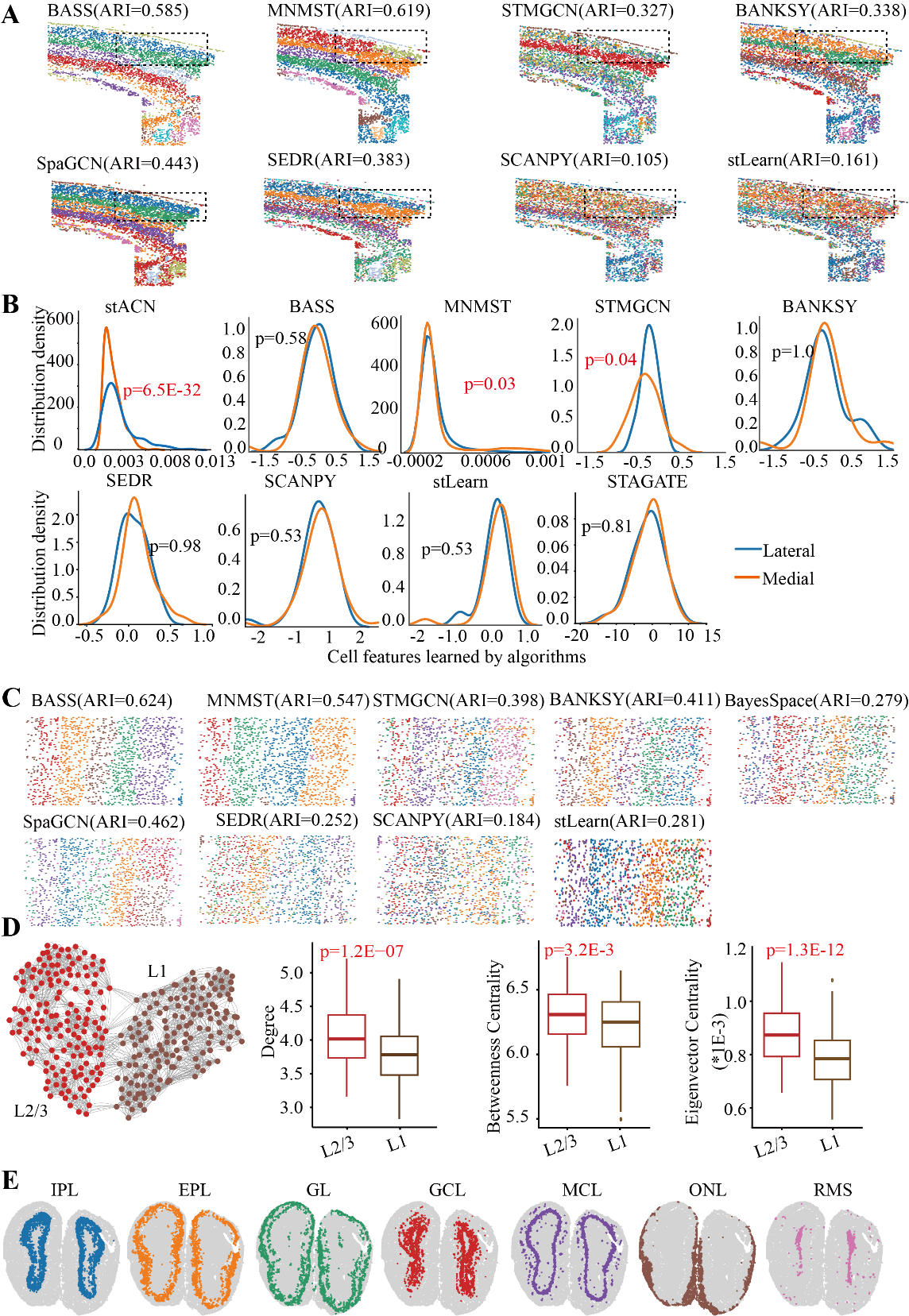


**Fig Q.** (**A**) Spatial domains identified by various algorithms on osmFISH SRT dataset. (**B**) Distribution density estimation among Lateral and Medial layers with features of cells learned by various algorithms (Kolmogorov-Smirnov test). (**C**) Visualization of spatial domains identified by various algorithms on STARmap dataset. (**D**) Topological structure of sub-networks induced by the L1 and L2/3 domain (left), and distribution of degrees, betweenness and eigenvector of cells in the Ll and L2/3 domain (Student's t-test for significance, right). (**E**) Scatter plot of spatial domains identified by stACN on Stereo-seq SRT dataset.


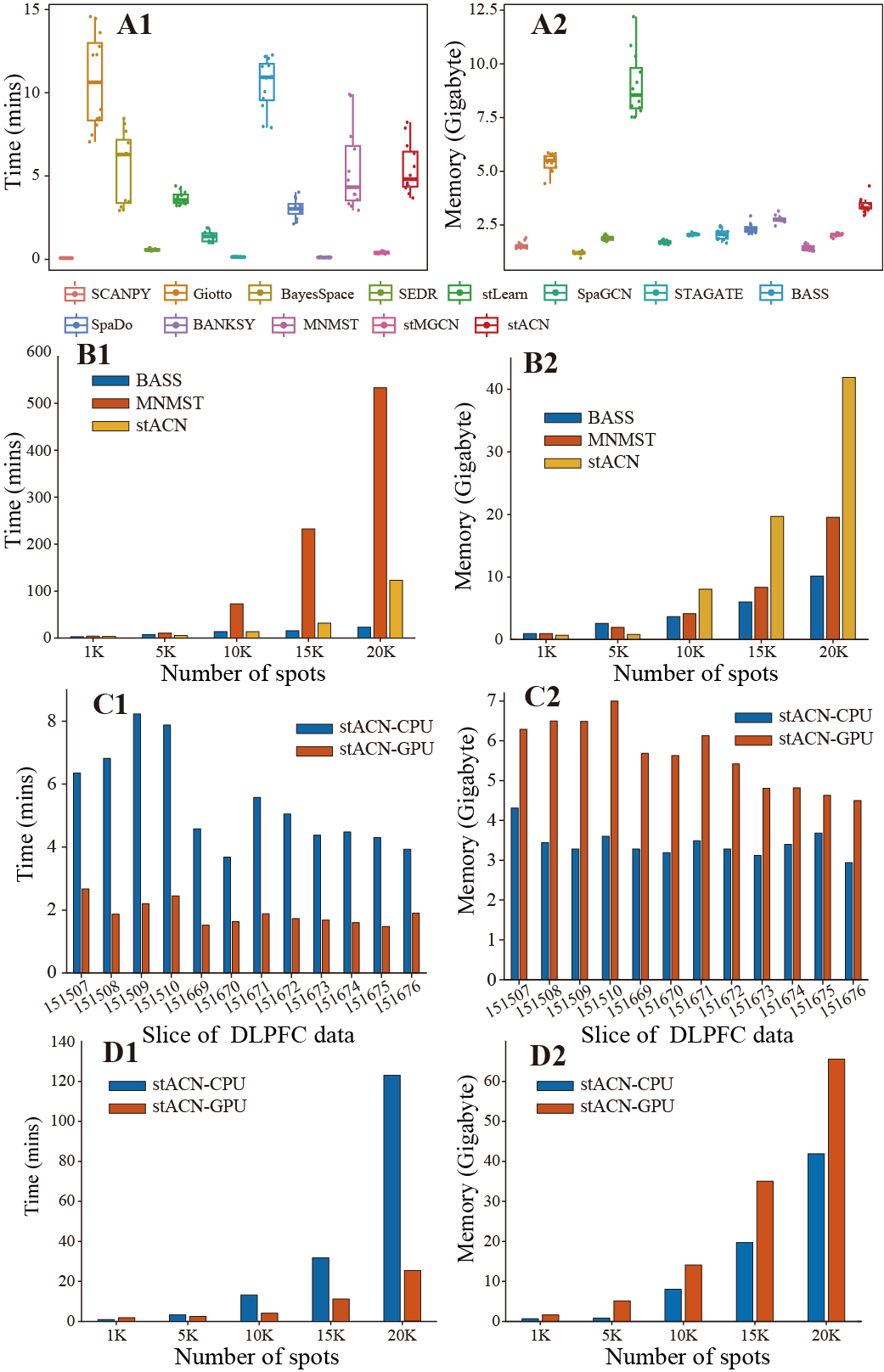


**Fig R.** Running time and space of algorithms for different spatial transcriptomics data, where missing bars represents out of memory. (**A1/A2**) Distributions of running time (minutes) and space (Gigabyte) of algorithms on the DLPFC data. (**B1/B2**) Running time and space of algorithms on MERFISH data with various sizes. (**C1/C2**) Running time and space of stACN with and without acceleration on the DLPFC data. (**D1/D2**) Running time and space of stACNwith and without acceleration on the MERFISH data.


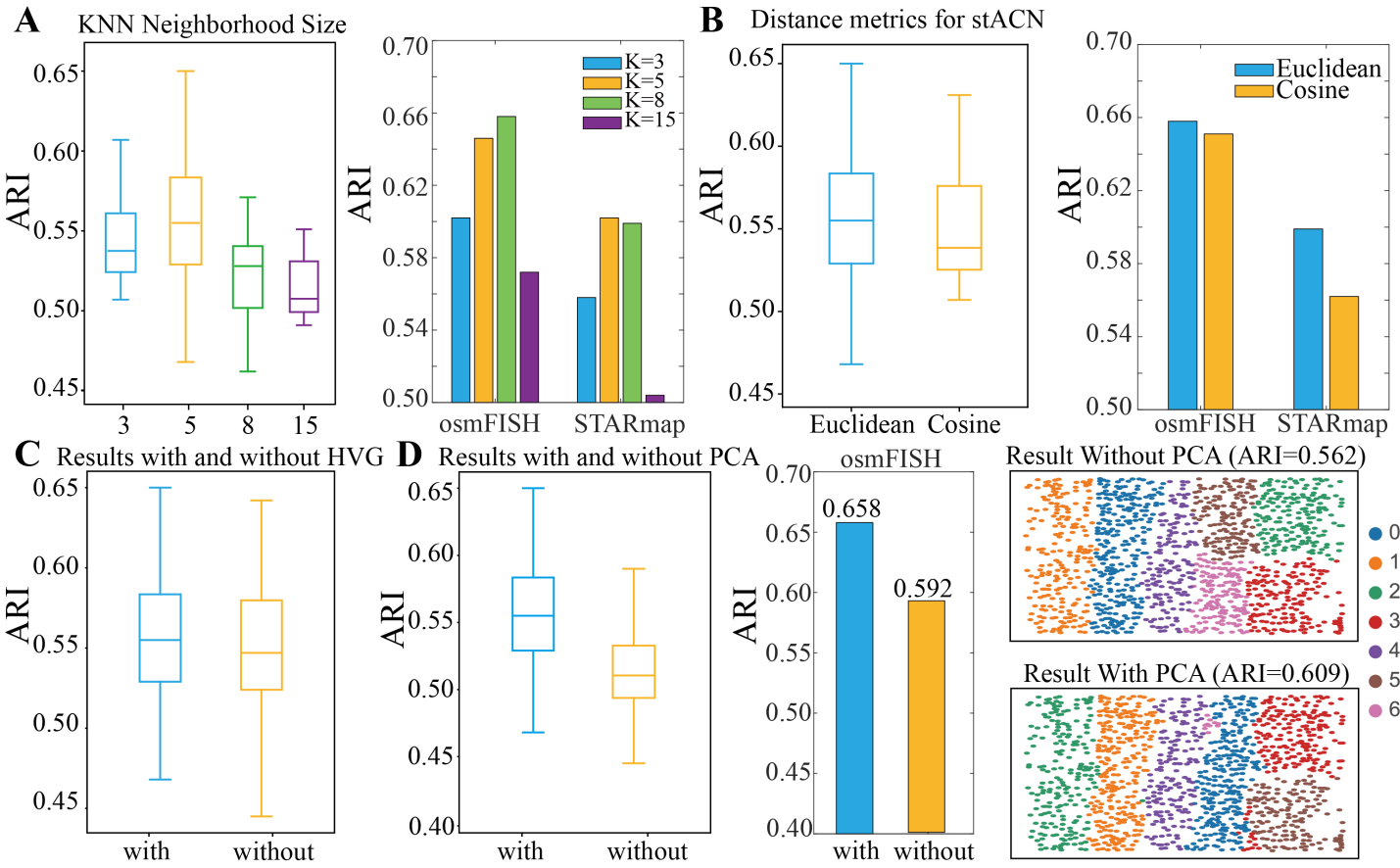


**Fig S.** Sensitivity and Robustness Analysis of stACN Pre-processing Settings. (**A**) Clustering performance across different KNN neighborhood sizes (K=3,5,8,15) for DLPFC, osmFISH, and STARmap datasets. (**B**) Comparison of Euclidean and cosine distance metrics for DLPFC, osmFISH, and STARmap datasets. (**C**) Distributions of ARIs with and without highly variable gene (HVG) selection for the DLPFC dataset. (**D**) Impact of PCA pre-processing on ARI for DLPFC (left), osmFISH (middle), and STARmap (right) datasets.
